# Intra- vs Intermolecular Cross-Links in Poly(methyl methacrylate) Networks Containing Enamine Bonds

**DOI:** 10.1021/acs.macromol.1c02607

**Published:** 2022-04-26

**Authors:** Soheil Sharifi, Isabel Asenjo-Sanz, José A. Pomposo, Angel Alegria

**Affiliations:** † Centro de Física de Materiales (CSIC, UPV/EHU)-Materials Physics Center (MPC), Paseo Manuel de Lardizabal 5, 20018 Donostia-San Sebastián, Spain; ‡ Department of Polymers and Advanced Materials: Physics, Chemistry and Technology, University of the Basque Country UPV/EHU, Paseo Manuel de Lardizabal 3, 20018 Donostia-San Sebastián, Spain; § IKERBASQUE-Basque Foundation for Science, Plaza de Euskadi 5, 48009 Bilbao, Spain

## Abstract

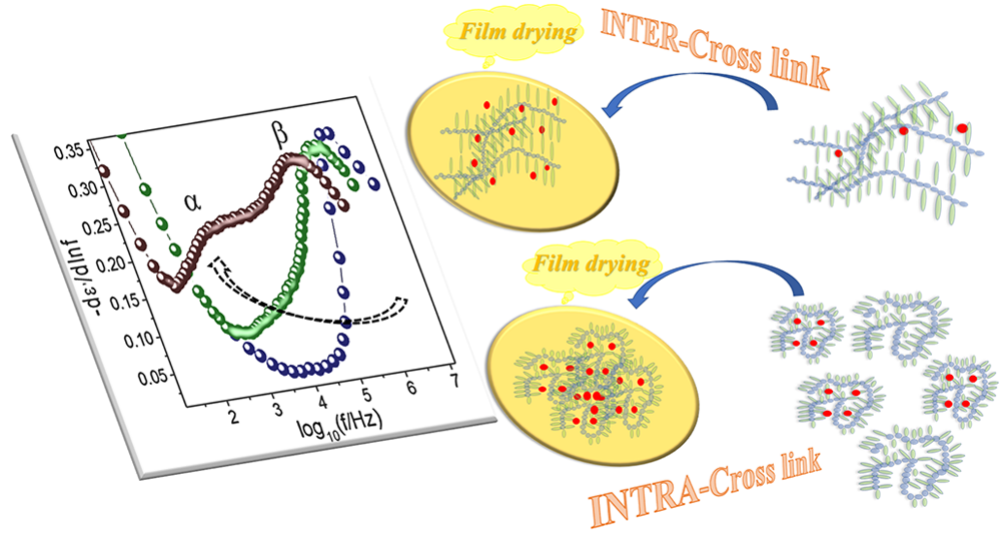

The molecular dynamics of a copolymer composed of methyl methacrylate (MMA) and (2-acetoacetoxy)ethyl methacrylate (AEMA) monomers and the influence on it of intra- to intermolecular cross-links of AEMA units with ethylenediamine (EDA) was studied by combining dielectric relaxation experiments and thermal investigations. The dielectric spectra of the non-cross-linked copolymer show three dynamical processes: a slow relaxation (α) and a faster (β), both dominated by the MMA dynamics, and an even faster secondary relaxation (γ) reflecting the AEMA dynamics. Already for low cross-linking densities, the γ process is very much affected and eventually disappears, increasing the cross-linking density. The secondary β relaxation however was nearly unaffected by cross-linking. The effect of cross-linking on the α relaxation was very pronounced with an important increasing of the glass transition temperature *T*_g_. There was also an increase of the dynamic heterogeneity and the relaxation intensity when increasing the cross-linking density (up to the maximum explored, 9 mol % EDA). The quality of the average time scale and *T*_g_ value have similarities in behavior for intra- and intermolecular cross-linking, but clear differences in the dynamic heterogeneities where observed. These differences can be interpreted in connection with the sparse internal structure of the collapsed single chains obtained by intramolecular cross-linking.

## 1 Introduction

The cross-linking of polymers has long been employed to change the physical properties of these materials such as rigidity and glass transition. Cross-linking is the production of covalent (permanent) or noncovalent (reversible) bonds, short sequences of connections, or physical interactions that hold together parts of a polymer chain (intrachain cross-linking) or different polymer chains (interchain cross-linking).^1−4^ Hydrogen-bonding, hydrophobic, and ionic interactions are for instance considered as noncovalent bonds, which can be reversibly activated or deactivated, while covalent (e.g., carbon–carbon) bonds are permanent bonds.^5,6^ In general, the polymers after cross-linking by covalent bonds cannot melt and only decompose by heating at very high temperatures.

Cross-linking is not limited to bulk materials, but also includes nano- to microsized particles with well-defined shape and size that eventually after cross-linking can be used, for example, as stable nanocontainers for drug delivery.^7,8^ Depending on the number of polymer chains involved in the cross-linking process, two types of cross-linking can be observed using a given polymeric material. When the cross-linking reaction is performed in dilute situations below the overlap concentration, intrachain cross-linking mainly occurs connecting distinct parts of every discrete single polymer chain, consequently collapsing the chain and reducing the hydrodynamic radius of the individual chains.^9^ The type of nano-objects obtained via intrachain cross-linking is usually referred to as single-chain nanoparticles (SCNPs). By increasing the concentration above the overlap concentration, interchain cross-linking can occur once two or more polymer chains are held together, and a competition between the two types of cross-linking happens. Finally, in very concentrated solutions (and in bulk) interchain cross-linking dominates over intrachain cross-linking, giving rise to 3D polymer networks.

The idea of producing discrete intrachain cross-linked polymer chains was suggested theoretically by Kuhn (1956), and it was done experimentally in 1962.^10,11^ However, it not was until the beginning of the 21th century that the potential of intrachain cross-linking to produce versatile SCNPs was identified.^12^ Nowadays, SCNPS can be routinely produced through a variety of different covalent, noncovalent, and dynamically covalent bonds.^13^ The size and surface-to-volume ratio of SCNPs can be controlled by the cross-linking density, the polymer nature and molecular weight, chain stiffness, etc.^14−16^ For instance, increasing the cross-linking density reduces the size of SCNPs, which offers unique benefits in medical applications.^8,7^ The majority of earlier SCNP synthesis research focused on irreversible bonds. Producing SCNPs that respond to external stimuli via dynamic covalent bonds has become a new topic in the field of smart materials.^8,17^ The breaking and exchange of these bonds can be induced by environmental effects, such as changes in temperature, pH, and solvent type.

Cross-linking reactions have an obvious impact on polymer chain mobility. Whereas interchain cross-linking will prevent the viscous flow, intrachain cross-linking giving rise to SCNPs yields a reduction of viscosity due to the decreasing of chain entanglements in the polymer melt.^18,19^ Despite this disparity of effects of intra- and interchain cross-linking on the large-scale polymer dynamics, both impose constraints at the more local scale like that of the segmental motions controlling the polymer glass transition (*T*_g_). What is generally observed is that as the cross-linking density increases, the polymeric material presents a higher *T*_g_,^20^ which in addition is broader and less pronounced. These effects are quite dramatic when the cross-linking density reaches large values,^21,22^ whereas they are no so large in the case of SCNPs when the cross-linking density is usually moderate. The investigations of the effect of intramolecular cross-linking on the glass transition of SCNPs are scarce,^23^ and there are few systematic studies on this aspect.^23^ In particular, a recent work combining experiments and simulation results on polystyrene (PS)-based SCNPs has shown a linear increase of *T*_g_ with the cross-linking density over a relatively broad range with a concomitant increase of the dynamic fragility and dynamic heterogeneity. These effects were interpreted in terms of the influence of the topological constraints present in the SCNPs. Although there is also a recent simulation work comparing the influence of intra- and intermolecular cross-linking on the structure and dynamics of polymer chains in solution,^24^ a systematic comparison of the evolution from intra- to intermolecular cross-links in networks based on SCNPs with dynamic covalent bonds is lacking.

With the aim of comparing in detail the effects on the segmental dynamics of “mostly” intra- and “mostly” interchain cross-linking, in this work, the evolution of the molecular dynamics and thermal properties of networks with dynamic covalent bonds prepared, on one hand, from SCNPs and, on the other hand, from the precursor polymer of the SCNPs were studied by broad-band dielectric spectroscopy (BDS) and differential scanning calorimetry (DSC). For this purpose, a random copolymer of methyl methacrylate (MMA), 70 mol %, and (2-acetoacetoxy)ethyl methacrylate (AEMA), 30 mol %, denoted as poly(MMA_0.7_-ran-AEMA_0.3_), was first synthesized. The cross-linking reactions to give SCNPs were performed in high-dilution conditions by reaction of ethylenediamine (EDA) as bifunctional cross-linker with the AEMA side groups of poly(MMA_0.7_-ran-AEMA_0.3_)—via enamine bond formation (see ref (25) and the SI)—reaching a maximum cross-linking density for 9 mol % EDA. In this way the effect of the intramolecular cross-linking on the segmental and local dynamics was detected with high detail. Similar experiments on equivalent materials prepared by performing the reactions in concentrated solutions promoting interchain reactions have been used to address the similarities and differences between the materials obtained from SCNPs and those obtained directly from poly(MMA_0.7_-ran-AEMA_0.3_) using more conventional interchain cross-linking procedures. The obtained results are discussed in comparison with previous experimental findings and recent molecular dynamics simulations.

## 2 Experimental Section

### 2.1 Materials

2,2-Azobis(2-methylpropionitrile) (AIBN), ethylenediamine, methyl methacrylate, 2-(acetoacetoxy)ethyl methacrylate, ethyl acetate (EtOAc), methanol (MeOH), tetrahydrofuran (THF), and deuterated chloroform (CDCl_3_) were provided by Merck. 2-Cyanoprop-2-yl-dithiobenzoate (CPDB) was supplied by Strem.

### 2.2 Synthesis of Poly(MMA_0.7_-ran-AEMA_0.3_)

In a typical procedure, MMA (1 mL, 9.4 mmol), AEMA (0.6 mL, 3.1 mmol), CPDB (6.9 mg, 3.1 × 10^–2^ mmol), and AIBN (1.3 mg, 7.8 × 10^–2^ mmol) were dissolved in EtOAc (3.2 mL). The solution was degassed by bubbling N_2_ for 15 min. The copolymerization reaction was carried at 65 °C for 18 h. After isolation of the resulting copolymer by precipitation in MeOH, it was dried under dynamic vacuum until constant weight.

### 2.3 Cross-Linking Reactions

**Scheme 1:**
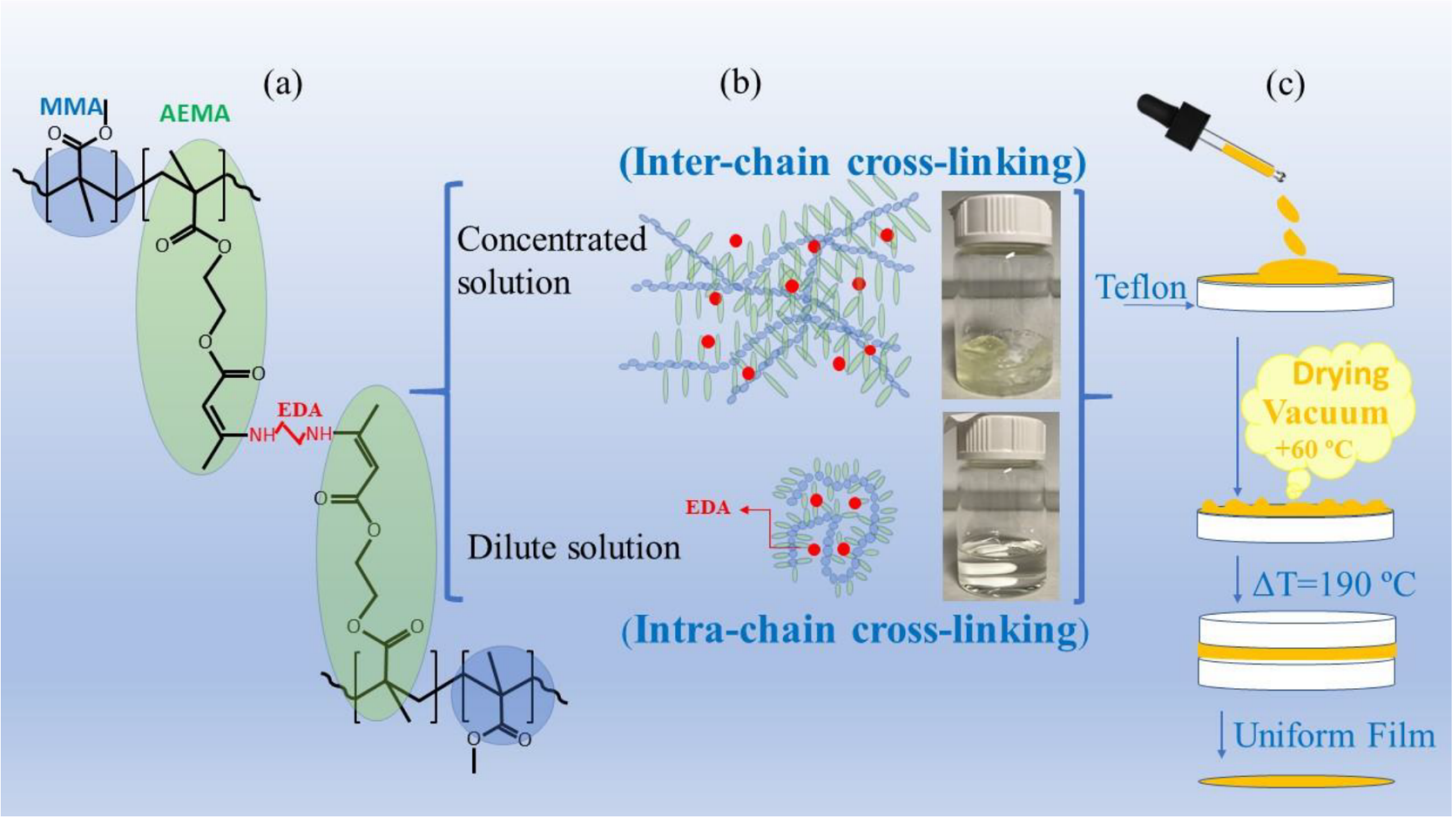
(a) Chemical Structure of the Cross-Linking between AEMA Units by Ethylenediamine (EDA) via Enamine Bond Formation (see ref (25) and the SI); (b) Preparation of THF Solutions above and below the Overlap Concentration for Inter- and Intra-Chain Cross-Linking, Respectively; (c) Procedure of Film Preparation from the above Solutions

Intramolecular cross-linked SCNPs were prepared from solutions below the overlap concentration by mixing poly(MMA_0.7_-ran-AEMA_0.3_) (100 mg, dissolved in 9.7 mL of THF) with respectively 0.032, 0.057, and 0.19 mL of the cross-linker solution (EDA, 2% in THF) at room temperature (rt). After reaction completion (20 h) (see Scheme 1), each solution was poured dropwise onto a Teflon sheet letting the solvent evaporate at rt and drying the remaining solid material under vacuum at 60 °C overnight. The resulting cross-linked copolymers—denoted as INTRA9, INTRA15, and INTRA50, respectively—were prepared in film form from the dried materials by hot pressing at 190 °C (Scheme 1). Networks based on intermolecular cross-linked poly(MMA_0.7_-ran-AEMA_0.3_) were prepared from solutions by mixing well above the overlap concentration (200 mg, dissolved in 1.94 mL of THF) by mixing poly(MMA_0.7_-ran-AEMA_0.3_) with respectively 0.115 and 0.381 mL of the cross-linker solution (EDA, 2% in THF) at rt. The final networks—denoted as INTER15 and INTER50, respectively—were then prepared following a procedure identical to that described for intramolecular cross-linked SCNPs (Scheme 1).

### 2.4 Characterization Techniques

DSC was carried out in a Q2000 TMDSC instrument. He gas with a flow rate of 25 mL/min was used in the sample area. Indium melting was used for calibration of the temperature and heat flow. Samples (about 5 mg) were encapsulated in aluminum containers of about 50 mg. The samples were first heated until 190 °C, kept for 3 min, and then cooled at 3 °C/min to 7 °C, using a modulation period of 60 s with 0.5 °C of modulation amplitude. The dielectric experiments were done using an ALPHA impedance analyzer from Novocontrol Technologies, covering a frequency range from 10^–1^ to 10^6^ Hz. The isothermal experiments were performed from 172 to −143 °C every 5 °C. Samples were located between two electrodes (10 mm diameter) of a parallel plate capacitor, and its temperature was controlled by a cryostat (BDS 1100) exposed to a heated gas stream evaporated from a liquid nitrogen dewar. The temperature control was assured by the Novocontrol Quatro Cryosystem and performed within ±0.2 °C.

## 3 Results

### 3.1 Characterization of Poly(MMA_0.7_-ran-AEMA_0.3_)

**Figure 1:**
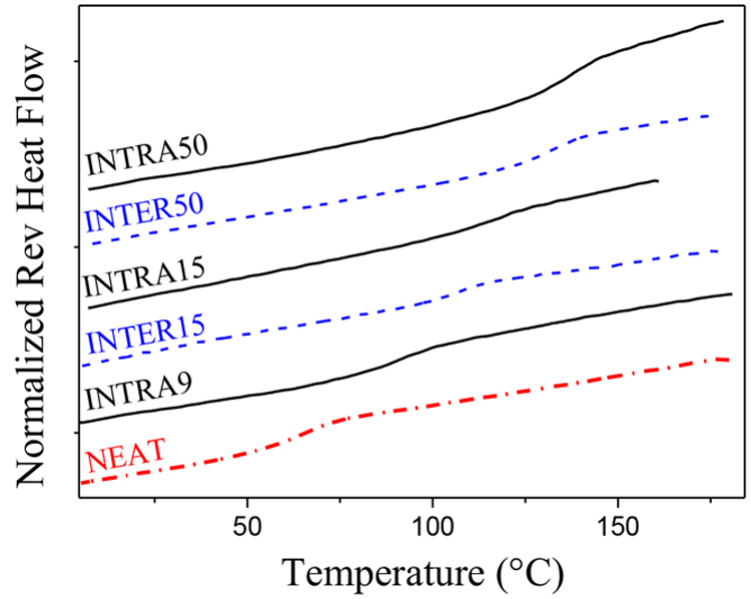
DSC heat flow curves of the samples investigated in this work as measured during cooling at 3 °C/min: neat poly(MMA_0.7_-ran-AEMA_0.3_) (denoted as NEAT, dotted-dashed line), networks based on intramolecular cross-linked SCNPs (denoted as INTRAx, solid lines), and networks based on intermolecular cross-linked poly(MMA_0.7_-ran-AEMA_0.3_) chains (denoted as INTERx, dashed lines) prepared with different EDA/AEMA molar ratios, from *x* = 9% to 50%.

The ^1^H NMR spectrum of poly(MMA_0.7_-ran-AEMA_0.3_) in CDCl_3_ confirmed the expected chemical structure of the copolymer, resulting in an MMA fraction in the copolymer of 70 mol % (see the SI). Concerning the molar mass, a weight-average molecular weight of *M*_w_ = 63.1 kDa, with a dispersity of *Đ* = 1.1, was determined by GPC (see the SI). The DSC trace (see Figure 1) shows a single step-like increment characteristic of a glass transition (*T*_g_), as expected for a random copolymer, located at around 63 °C (see Figure 1, dotted-dashed line), which is a temperature significantly lower than the *T*_g_ of atactic PMMA (105 °C).

### 3.2 Dielectric Study of Poly(MMA_0.7_-ran-AEMA_0.3_)

**Figure 2:**
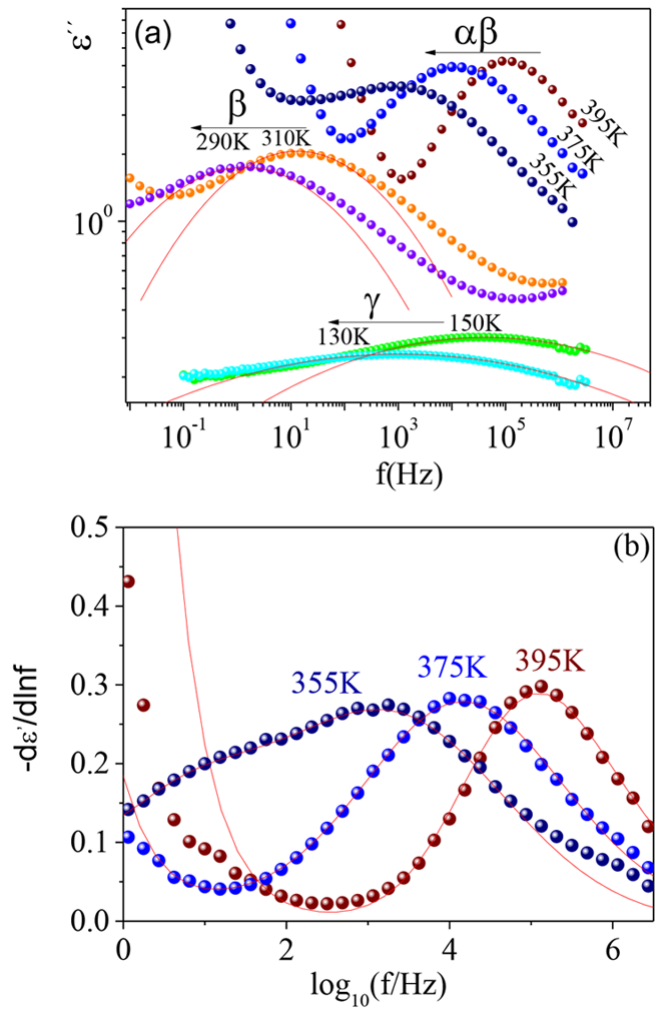
(a) Dielectric permittivity losses (ε″) as function of frequency (*f*) for the neat poly(MMA_0.7_-ran-AEMA_0.3_) copolymer at different temperatures. (b) Experimental −dε′(*f*)/d ln *f* as function of log_10_*f* at three representative temperatures in the merging region and corresponding fitting lines (see text for details).

The detailed molecular dynamic processes of the neat poly(MMA_0.7_-ran-AEMA_0.3_) copolymer were investigated by studying the dielectric relaxation behavior of this material. Representative dielectric loss spectra obtained isothermally, on progressively cooling the sample from 142 °C, are shown in Figure 2.

Below *T*_g_, two local relaxation processes (β- and γ-process) were observed. The β-process origin is the rotation of the −COOCH_3_ side groups of MMA repeating units since it compares quite well with that observed in syndiotactic PMMA.^26^ As PMMA does not show a remarkable dielectric γ-relaxation process, the origin of the γ-process observed for the copolymer must be the molecular motions of the AEMA repeating units, particularly from the quite polar side group containing the β-ketoester moiety. The relaxation times of the β- and γ-processes were obtained at each temperature from the corresponding dielectric loss peak frequency via τ = (2π*f*_max_)^*–*1^.

**Figure 3:**
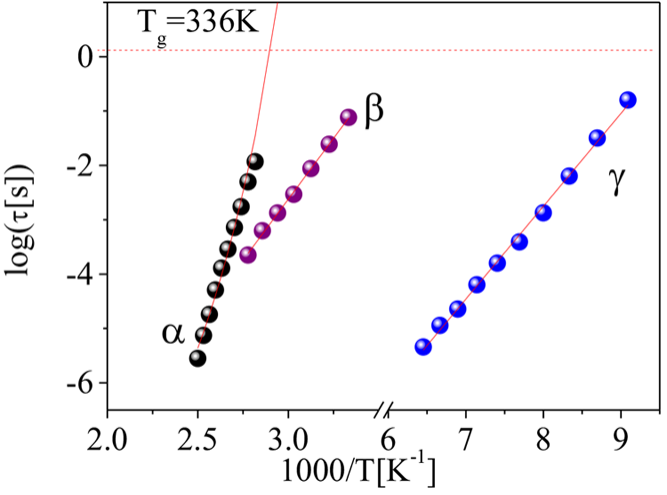
Relaxation map of the poly(MMA_0.7_-ran-AEMA_0.3_) copolymer. The solid lines are the fitting using eq 6 for α-relaxation and the Eyring equation for the β- and γ-relaxations (see text for details).

As can be seen in Figure 3, the temperature dependences of τ_β_ and τ_γ_ are quite linear in the Arrhenius representation, as expected for localized molecular motions. It can be noted that the relaxation times of the β-process in the copolymer are nearly identical to those found in the secondary relaxation of pure PMMA.^27−29^ Both temperature dependences can be well fitted with the Eyring equation:
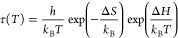
1where *k*_B_ and *h* are the Boltzmann and Plank constants (*k*_B_*T*/*h* is the Debye frequency) and Δ*S* and Δ*H* are the activation entropy and the activation enthalpy, respectively. The former is related to the cooperativity degree and the latter to the activation barrier.^30^

The values of the corresponding activation enthalpies and activation entropies are respectively 82.9 kJ/mol and 59.2 J/K/mol for the β-relaxation, while they are 28.8 kJ/mol and 56.7 J/K/mol for γ-relaxation. The obtained results showed a much lower barrier for the γ-relaxation than that for the β-relaxation, but both processes show a comparable cooperativity. A characteristic temperature can be obtained in each case form the condition *T*_C_ = Δ*H*/Δ*S*. At *T*_C_ the relaxation time of the process would reach the value corresponding to the reciprocal of the Debye frequency. From *T*_C_ = Δ*H*/Δ*S*, we obtain *T*_C_ = 1400 K for the β-relaxation and *T*_C_ = 508 K for the γ-relaxation.

The obtained values of the activation enthalpy and entropy for each secondary relaxation are the average of a distribution, since these local motions occur in the glassy state where the molecular environment is far from being homogeneous. We have found that a Gaussian distribution of the activation enthalpy, *G*(Δ*H*), provides a good approach for the experimental data by assuming that the *T*_C_ value remains unchanged; that is, there exists a corresponding Gaussian distributions of activation entropies. Within this framework, the dielectric losses can be described as a superposition of single relaxation processes each characterized by an activation enthalpy Δ*H* and an activation entropy Δ*S* = Δ*H*/*T*_C_, i.e.,^27,31−33^
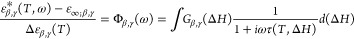
2where Δε indicates the strength of the dielectric relaxation, ε_*∞*_ is the high-frequency limiting permittivity, and the values of τ(*T*, Δ*H*) are calculated by means of eq 1. Figure 2(a) shows some representative curves obtained in this way, which account not only for the peak position but also for the relaxation loss peak shape around the peak. The standard deviation of the corresponding *G*(Δ*H*) distributions was σ = 11.6 kJ/mol for the β-relaxation and σ = 14.4 kJ/mol for the γ-relaxation.

The secondary relaxation strength values of the neat poly(MMA_0.7_-ran-AEMA_0.3_) copolymer were also obtained in this way. The Δε_β_ (β-process) value changed moderately from 1.7 to 1.9 with increasing the temperature from 275 K to 315 K, whereas the Δε_γ_ (γ-process) value was nearly constant (Δε_γ_ = 0.48) at all temperatures investigated.

Above *T*_*g*_, the α-relaxation of poly(MMA_0.7_-ran-AEMA_0.3_) merges with the β-process, in a way similar to that found for pure PMMA, making the analysis more complicated. The data analysis is further made difficult by a significant conductivity-related contribution at the lowest frequencies. A way to overcome this latter difficulty is to analyze the derivative of the real part, −dε′/d ln *f* (see Figure 2b), where the dc conductivity does not contribute. As can be seen in Figure 2b, in this way the dielectric losses from the α-relaxation are quite clearly distinguishable in the low-frequency flank of the experimental relaxation peak at relatively low temperatures. The remaining increasing contribution at lower frequencies corresponds to interfacial phenomena associated with several factors, for instance, sample inhomogeneities and bubbles, and also with electrode polarization. It is also clear from the data of Figure 2 that the contribution from the α- and β-relaxation overlaps more as the temperature increases. This merging phenomenon has been explored by using the so-called Williams ansatz,^34^ in which it is considered that the same molecular dipoles contributing to the secondary relaxation, by limited angular reorientations, become able to fully reorient as far as the sample temperature overtakes *T*_g_. In this framework, the dielectric relaxation function can be expressed as
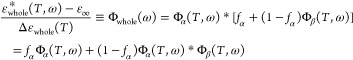
3where * represents a convolution product and *f*_α_ is the relative contribution of the α-process to the whole dielectric relaxation strength Δε = ε*_whole_(ω → 0) – ε_*∞*_.^27^

In general, at relatively low temperature, the α- and β-processes are well separated and Φ_α_(ω) and Φ_β_(ω) can be determined independently. This is particularly the case for Φ_β_(ω, *T*) below *T*_g_. In the merging region, the behavior of Φ_β_(ω, *T*) can be extrapolated from low temperatures by considering that the description of the secondary relaxation found below *T*_g_ remains valid also above *T*_g_. Thus, the fitting of the experimental data in the merging region will provide the information about the α-relaxation. A convenient way to compute the convolution product in eq 3 is by describing the α-relaxation also in terms of a distribution of Debye-like relaxation functions. Thus, in order to account for the α-relaxation components, we have used the following distribution of relaxation times:^27^
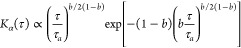
4where the two parameters are τ_a_, a characteristic time, and *b*, a parameter determining the shape of the distribution. Interestingly, for *b* = 0.5 the resulting relaxation function corresponds to a Kohlrausch-Williams-Watts (KWW) time decay with β = 0.5 and τ_KWW_ = τ_a_.^27^ Note that the KWW (stretched-exponential) relaxation function is a quite common way to account for cooperative relaxation processes like that associated with the main chain backbone motions originating the α-relaxation. Within this framework the whole relaxation function eq 3 can be expressed as
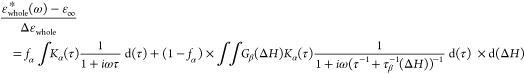
5

Note that the effective time scale in the second term is close to the fastest relaxation component, which at low temperature is that of the β-relaxation, but not always in the merging region. An additional power law contribution was required to account for the lowest frequency increasing. The lines describing the data in Figure 2b were calculated in this way, resulting in *b* values ranging from 0.41 to 0.5 at the highest analyzed temperatures. The α-relaxation peak times, τ_α_(*T*), were calculated from the distribution parameters τ_α_ and *b* numerically as the reciprocal frequency at the maximum, τ_α_ = (2π*f*_max_)^−1^, of the resulting loss peak. The fitting procedure provides also the strength of the whole relaxation function and the corresponding fraction *f*_α_. These α-relaxation time values (see Figure 3) follow a non-Arrhenius behavior that can be described with the Vogel-Fulcher (VF) function
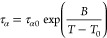
6where *B* and *T*_0_ are fitting parameters and τ_α0_ was assumed equal to 0.1 ps, a typical time of molecular vibrations. In this way we obtained *B* = 2815 K and *T*_0_ = 244 K. So, at the glass transition temperature as determined by calorimetry (336 K) the resulting relaxation time is τ_α_(*T*_g_) = 1.95 s.

### 3.3 Effect of Intrachain Cross-Linking

**Figure 4:**
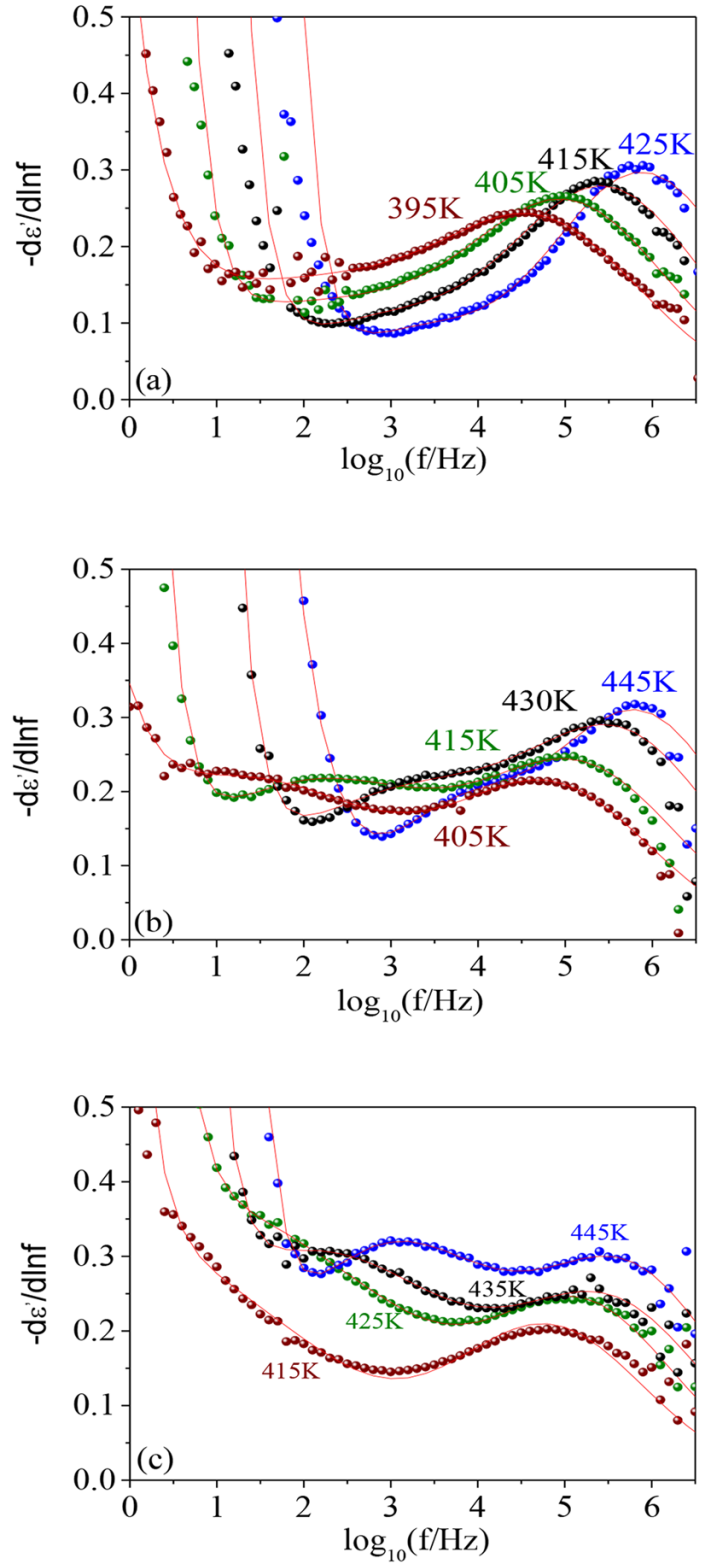
Spectra of −dε′(*f*)/d ln *f* as function of log *f* for (a) INTRA9, (b) INTRA15, and (c) INTRA50. Continuous lines are fits by the model function (see text for details).

Intramolecular cross-linking of poly(MMA_0.7_-ran-AEMA_0.3_) was produced at high dilution conditions with the addition of different EDA amounts to reach EDA/AEMA molar ratios in the range from 9% to 50%. The resulting materials were heated to 190 °C after completely drying the solutions under vacuum at 87 °C. Comparison of infrared absorption data with those of the copolymer confirmed the reaction of EDA with the β-ketoester moiety of the AEMA units (see the SI). The increasing cross-linking density results in a significant increase of the glass transition temperature (see Figure 1), up to 146 °C for the highest cross-linking density, which reflects the additional constraints to the segmental scale mobility imposed by the dynamic enamine bonds. This was also very clearly reflected in the dielectric relaxation behavior. Figure 4 shows representative results.

There are several effects of the EDA addition in the dielectric relaxation of the cross-linked materials based on intramolecular cross-linked SCNPs. Concerning the local relaxation processes, the situation is odd. On the one hand, the characteristics of the β-relaxation are not significantly affected beyond the relaxation strength, although we detected minor changes in the average activation enthalpy (see the SI). On the contrary, the γ-relaxation is dramatically suppressed and essentially disappears for ratios higher than 9%. This confirms both the assignment of the relaxation to motions of moieties within the AEMA units and the reaction of EDA with the β-ketoester moieties. The effect on the α-relaxation is also important. First, the relaxation becomes slower, as could be anticipated by the higher *T*_g_ values. This is one of the reasons that the dielectric α-relaxation is less overlapped with the β-relaxation. Also, there seems to be an important increase of the relaxation strength, which could be partially related with the new dipolar entities generated during the cross-linking reaction but also with the blocking of unreacted AEMA units that would become able to reorient only at higher temperatures. To study these changes in detail, we analyzed the relaxation behavior of the cross-linked materials using an approach similar to that used for the copolymer. The major difference is that we considered that cross-linking induces additional dynamic heterogeneities by modifying the distribution function characterizing the α-relaxation. Particularly, a new distribution *K*_α,cross_(τ) was introduced, which results from the convolution of *K*_α_(τ) with a Gaussian distribution of the corresponding characteristic times. This Gaussian distribution would account for the “extra” heterogeneities induced by the cross-links:
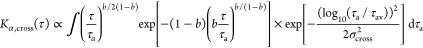
7

**Figure 5:**
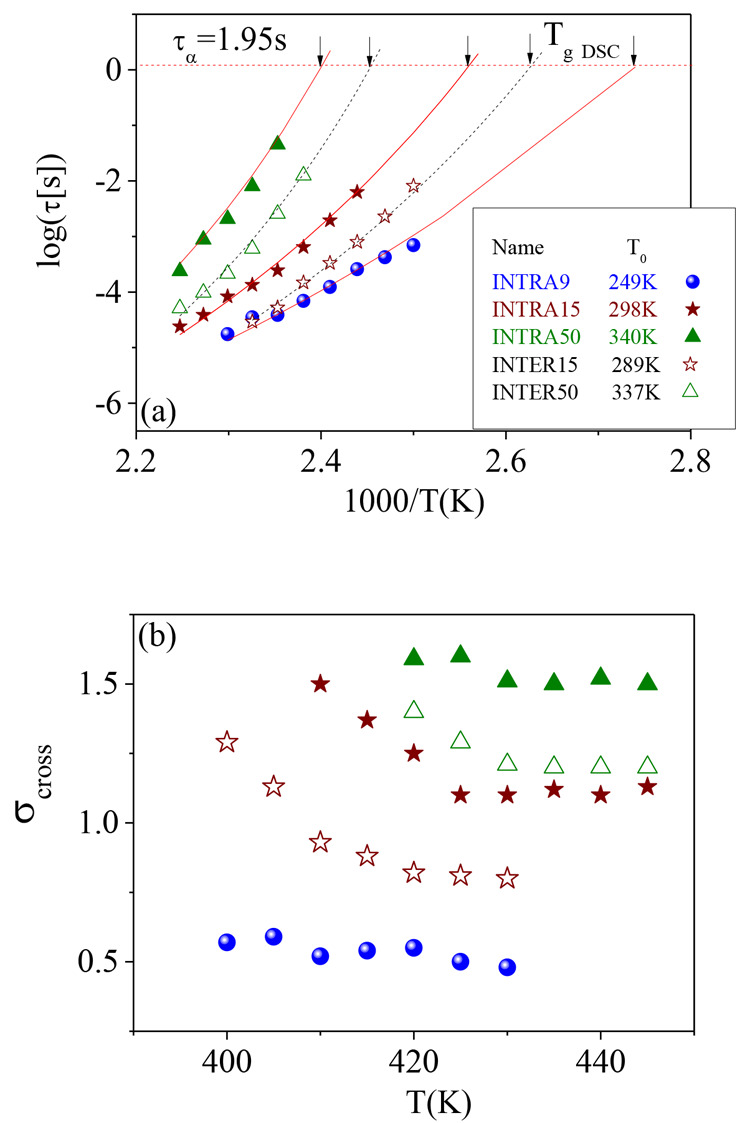
(a) Relaxation map of INTRA9 (solid square points), INTRA15 (solid star points), INTRA50 (solid up-triangle points), INTER15 (open star points), and INTER50 (open up-triangle points). (b) σ_cross_ parameter as a function of temperature for different cross-linking density (same symbols as in (a)).

Note that by using σ_cross_ = 0, eq 7 reduces to eq 4 and therefore corresponds to the non-cross-linked copolymer response, since the Gaussian factor becomes zero valued except for τ_a_ = τ_av_. In order to maintain the number of fitting parameters the same and taking into account that the relaxation in the cross-linked copolymers is analyzed at relatively high temperature, *b* = 0.5 was fixed. Lines in Figure 4 correspond to the fitting curves so obtained. Again, the α-relaxation peak times, τ_α_(*T*), were calculated numerically as the reciprocal frequency at the maximum of the resulting loss peak. These values together with the corresponding σ_cross_ ones are presented in Figure 5 (filled points). As could be expected, increasing the cross-linker fraction produces simultaneously a slower and more heterogeneous segmental dynamics.

To fit τ_α_(*T*) for cross-linked copolymers, first we fit the data of the INTRA50 sample (having the highest cross-linking density) in the same way as for the copolymer. In this way we found for this material the same value τ_α_(*T*_g_) = 1.95 s as for the neat copolymer, and therefore we decided to fix it for all the other materials; that is, it is possible to rewrite eq 6 as
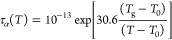
8

In this way, a single parameter is free (*T*_0_); see Figure 5(a) (and the inset in this figure). The *T*_0_ value increases from 244 K to 340 K with increasing the cross-linking density. The other important parameter in the fitting of the cross-linked materials is that accounting for the additional dynamical heterogeneities, which in the proposed description is captured by σ_cross_ measuring the breadth of the Gaussian distribution in eq 7. As can be seen in Figure 5(b), the σ_cross_ variation with *T* is weak at high temperature, where this parameter is determined with lower uncertainty. This plateau-like value changes from 0.5 to 1.5 with increasing the cross-linking density from INTRA9 to INTRA50.

### 3.4 Effect of Interchain Cross-Linking

**Figure 6:**
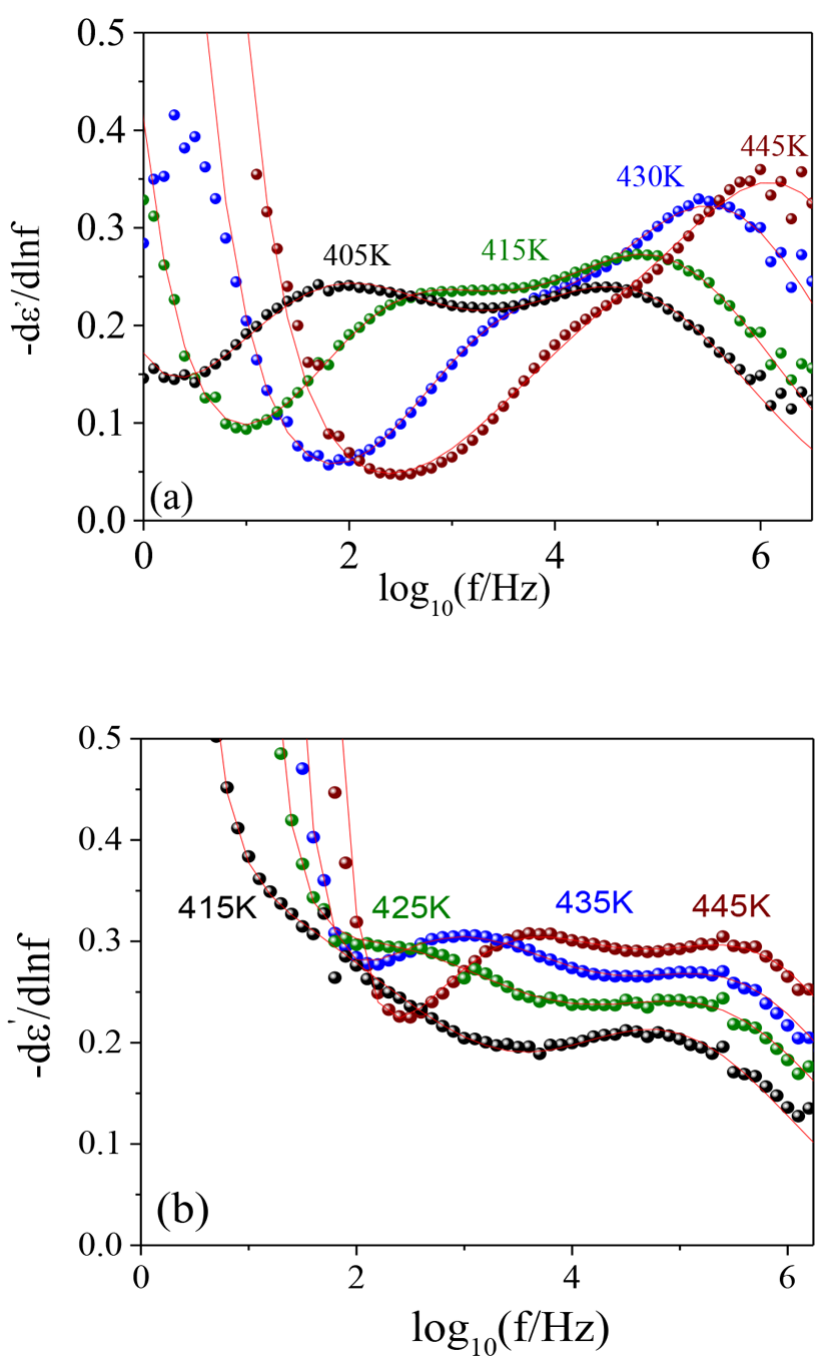
Spectra of −dε′(*f*)/d ln *f* as a function of log *f* for (a) INTER15 and (b) INTER50 at different temperatures. Continuous lines are fits by merging model and conductivity (σ) contributions.

Similar experiments were performed on samples where the cross-linking reaction took place in higher concentrated solutions (above the overlap concentration), which would favor the formation of interchain cross-links instead of SCNPs giving rise to a polymer network. Corresponding experimental results by DSC are presented in Figure 1 as dashed lines, whereas dielectric data are shown in Figure 6 and also in the SI. It is observed that also here the α- and β-processes are more separated with an increase in cross-linking density.

As for the materials cross-linked in dilute solution, eq 7 was used to fit the dielectric behavior to obtain the two main parameters characterizing the α-relaxation, τ_αv_ and σ_cross_, which are shown in Figure 5 (open points). The *T*_0_ value was also extracted from a fit of τ_α_(*T*) with eq 8, and the values of *T*_0_ were found to be 289 K for INTER15 and 337 K for INTER50. Moreover, also the values of σ_cross_ were obtained and were distinctly lower than those obtained for the corresponding material prepared from SCNPs.

## 4 Discussion

In the previous section, we have shown how the dielectric relaxation behavior of poly(MMA_0.7_-ran-AEMA_0.3_) is modified by the cross-linking reaction with EDA, both at high dilution (below the overlap concentration) and in more concentrated conditions (above the overlap concentration). In the former case, during the reactions, the intramolecular cross-linking would produce a partial collapse of the isolated polymer chains forming discrete SCNPs, which has been found to influence the polymer motions and particularly the segmental dynamics associated with the glass-transition phenomenon. The detailed study of these effects has been possible thanks to the high sensitivity of the dielectric relaxation to the mobility of the dipolar entities of the polymers. In the case of the neat poly(MMA_0.7_-ran-AEMA_0.3_) copolymer two distinct moieties are relevant, the ester group of the MMA repeating units and the β-ketoester group of the AEMA repeating units. These latter dipoles are in a relatively long side chain and are found to move to a large extent at very low temperatures, which resulted in the γ-relaxation observed in our experiments. In contrast, the ester group mobility is detected only close and above room temperature, in a manner that resembles very much the behavior observed in isotactic PMMA.^35^ Particularly, the time scale and activation energy of the β-relaxation of PMMA nearly coincide with that observed in the copolymer, which evidence the major intramolecular character of the interactions controlling the mobility of the ester group below *T*_g_. This is further confirmed by the fact that the characteristics of the ester local-mobility were essentially unaffected by the cross-linking reactions. The situation with respect to the mobility of the AEMA side group is obviously different, since it is directly involved in the cross-linking reactions by establishing a link with another AEMA side group via enamine bond formation. This is the reason that the prominent dielectric γ-relaxation observed at low temperatures in the copolymer nearly vanishes already for both INTRA15 and INTER15 materials (see the SI). Note that the cross-linking reactions also introduce new dipolar entities with a quite restricted mobility since they will be located at the cross-linking points. Nevertheless, one could expect detecting the motion of such newly introduced dipoles in the analysis of the α-relaxation. In the copolymer, the dielectric α-process from the main chain backbone motions presents a relatively weak amplitude and appears combined with the β-process originated by the motion of the methyl ester, −COOCH_3_, side groups. This fact produces a merging of the two relaxations (αβ-process) immediately above *T*_g_, which is even more important than in PMMA due to the *T*_g_ reduction caused by the incorporation in the copolymer of the AEMA units. The cross-linking reactions on the contrary promote the separation of these two processes, and the increase in α-process amplitude was evidently observed (see Figures 4 and 6). This higher intensity of the dielectric α-relaxation would result from the contribution of AEMA dipoles that are no longer able to reorient at low temperatures and newly created dipolar entities at the cross-linking points. Both will contribute to the dielectric relaxation only above *T*_g_.

**Figure 7:**
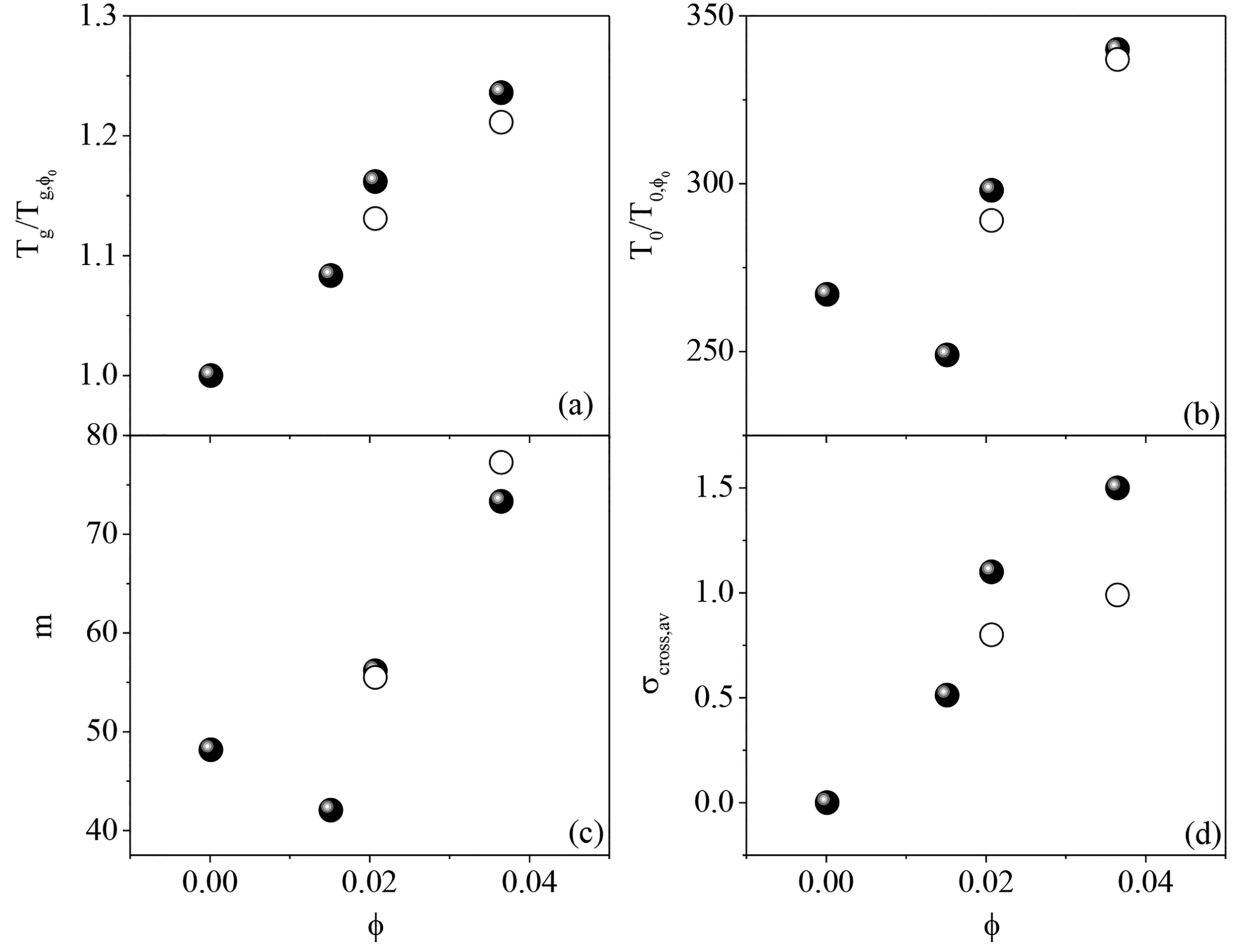
(a) *T*_g_ and (b) *T*_0_ of networks based on intramolecular cross-linked SCNPs (solid circles) and networks based on intermolecular cross-linked poly(MMA_0.7_-ran-AEMA_0.3_) chains (open circles) as a function of the cross-linking degree (ϕ) calculated as the relative number of repeating units involved in the reactions. The values are normalized by the parameters of the neat poly(MMA_0.7_-ran-AEMA_0.3_) copolymer. (c and d) Corresponding behavior of the fragility index *m* and the additional dynamic heterogeneity σ_cross_.

Also, in the previous section we have illustrated in detail how the resulting polymer segmental dynamics are affected by the introduction of a relatively low amount of cross-links. The effects were very clear even if the cross-linking degree (ϕ), calculated as the relative number of repeating units involved in the reactions, was in all cases below 0.1. To quantify the actual cross-linking degree values of the different samples, we have analyzed the infrared absorption bands in the range between 1560 and 1680 cm^–1^, a range where the neat copolymer does no show measurable absorption. As can be seen in the SI, the absorption intensities increase with increasing the EDA concentration. However, whereas the increase is approximately linear for low DEA amounts (below 15%), it tends to lower values for the highest DEA amount (50%). This behavior reflects the fact that at relatively large amounts of DEA the cross-linking reactions cannot be completed. If we assume that for the copolymer with 9% wt DEA all possible cross-linking reactions giving rise to enamine bonds took place, the ratio between the IR absorption signal (area of the absorption band) and the actual cross-linking degree would be 217 cm^–1^. In this way we have estimated the actual cross-linking point density from the IR absorption signal of the samples, obtaining a maximum value of ϕ = 0.036 for the INTRA50 sample. Once the cross-linking degree (ϕ) for every sample has been established, first, we will discuss the *T*_g_ changes. Figure 7a shows a linear behavior of glass transition, which is in line with that found on polystyrene-based SCNPs over a wider range of cross-linking densities, by both simulations and experiments.^24^ A distinct behavior is observed at low cross-linking densities when analyzing the Vogel temperature *T*_0_ (see Figure 7b). Here, a first decrease for low cross-linking degree is found, but *T*_0_ increases at higher concentrations. In addition, a similar trend is found by analyzing the so-called dynamic fragility, *m*, which is defined as^36,37^
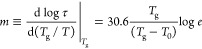
9

Also, *m* decreases first and then it increases in a nearly linear way. The nonmonotonous changes with the cross-linking degree (ϕ) of *T*_0_ and *m* could be attributed to the influence of the molecular dynamics involving the enamine bonds. In the neat copolymer, the segmental dynamics is dominated by the molecular dynamics involving MMA segments, whereas the fast AEMA side group motions have a plasticizer effect. Once the enamine cross-linking bonds are introduced, this is no longer the case since the molecular motions of MMA become coupled with those involving the intramolecularly cross-linked AEMA units. This is also manifested by the strong suppression of the dielectric γ-relaxation in the cross-linked materials.

Remarkably, in addition to the changes in the time scale of the polymer segmental dynamics, our analysis allowed quantifying the additional dynamic heterogeneity due to the cross-links. Figure 7(d) shows that σ_cross_ also increases in a linear fashion with cross-linking density, mirroring that of *T*_g_. Therefore, overall, our results suggest that the major effect of the intramolecular cross-links in the segmental dynamics is a slowing down of the segmental dynamics accompanied by an important increase of the dynamic heterogeneity.

The results discussed above refer to the materials obtained by cross-linking in highly dilute solutions, i.e., where interchain reactions are prevented, giving rise to intrachain linkages producing collapsed single chains referred to as SCNPs. For comparison, we also prepared cross-linked materials in relatively high concentration conditions, therefore promoting interchain reactions. We found that the segmental dynamics in these more conventional cross-linked polymers are quite similar to those found before. The differences in the time scales of the segmental dynamics are evident but quite small. The situation is however different with respect to the additional dynamic heterogeneities as quantified by σ_cross_; the σ_cross_ value of INTER50 is even lower than that of INTRA15, which indicates a clearly more homogeneous dynamics for the material prepared in concentrated solutions.

The remarkable similarities found above when comparing INTRA and INTER materials could be partially attributed to the dynamic character of the enamine cross-linking bonds, as demonstrated by the variety of vitrimers^38^ that have been prepared based on this “forgotten” dynamic covalent bond. Nevertheless, the clear differences in the broadness of the distribution characterizing the dynamic heterogeneities produced by cross-linking evidence that those materials prepared starting from the dilute solution of SCNP preserve a majority of intramolecular cross-links. Taking into account the crumpled globule morphology of the intramolecularly collapsed chains forming SCNP, it is clear that the distribution of cross-links in the resulting materials will be highly heterogeneous, and therefore segments with very restricted mobility will coexist with others remaining weakly affected by the cross-links. On the contrary, in the more conventional networks obtained from the cross-linking in concentrated solutions the cross-linking points are expected to be more uniformly distributed, and as a consequence the segmental motion rates will be less heterogeneous. The relatively short length scales (∼1 nm) involved in the segmental dynamics can explain why the behavior of the average time scale for the segmental dynamics is not so different when comparing intra- vs interchain cross-linked materials.

Finally, we comment on the reported connection between the cross-linking process and the free-volume reduction.^39^ From the above results we could conclude that the amount of free-volume reduction does not significantly depend on the inter- or intramolecular nature of the cross-links.

## 5 Conclusions

A systematic study of the effect of intramolecular cross-links giving rise to SCNPs on the molecular dynamics as studied by BDS and DSC was presented. These SCNPs were prepared based on a poly(MMA_0.7_-ran-AEMA_0.3_) copolymer and using EDA as cross-linker. The dynamics in the glassy state of the neat copolymer, as a reference, is manifested by the two dielectric relaxations, β- and γ-processes, detected by BDS. The Arrhenius-like temperature dependences of both characteristic times are described with the Eyring equation and the relaxation shapes accounted for on the basis of distributions of Debye-like relaxation functions. The same description of these localized molecular motions was considered to remain valid at higher temperatures. Above the glass transition temperature, the Williams’ ansatz model was used for describing the merging of the weak dielectric α-relaxation with the dielectrically stronger β-process. Different amounts of EDA were used as cross-linker to study the effects of enamine bonds on the molecular dynamics. It was observed that the α-relaxation times were separate from the nearly unaffected β-relaxation times with an increase of the cross-linking density. This was accompanied by a concomitant increase of the dynamic heterogeneities and also of the dynamic fragility. The same approach was used to analyze the materials obtained when the cross-linking reactions were promoted to be mainly of intermolecular character (reaction in solution above the overlap concentration). Qualitatively similar results were obtained in both cases, which was attributed to the relatively local character of the molecular motions investigated. Nevertheless, an evident more heterogeneous relaxation is observed in materials prepared by intrachain cross-links. This is an effect reflecting the crumpled globule morphology of the intramolecularly collapsed chains forming SCNPs where the number of cross-links is large in parts of the chains but scarce in others. For the materials prepared from higher concentration solutions the intermolecular cross-links will be promoted, giving rise to a more uniform cross-linking point distribution, a typical characteristic of conventional polymer networks.

## Supporting Information Available

Detailed experimental methods, including study of the enamine bond reversibility, proton NMR spectra for poly(MMA_0.7_-ran-AEMA_0.3_), size distribution of SCNPs with different cross-link degree, ATR-FTIR spectra of PMMA, AEMA, EDA, and poly(MMA_0.7_-ran-AEMA_0.3_) copolymer, ATR-FTIR absorbance of copolymer samples with different cross-link degree, dielectric results of the cross-linked copolymers, and full-scale GPC chromatograms (PDF)
