# Supplementary material for: Intra- vs Intermolecular Cross-Links in Poly(methyl methacrylate) Networks Containing Enamine Bonds
Source: Macromolecules. 2022 Apr 26;55(9):3627–36. doi: 10.1021/acs.macromol.1c02607 (PMC9100347; doi:10.1021/acs.macromol.1c02607)
Supplement: Supplementary file 1 — ma1c02607_si_001.pdf [file ma1c02607_si_001.pdf]

Supporting Information

Intra- vs Inter-Molecular Cross-Links in Poly(Methyl Methacrylate) Networks containing Enamine Bonds

Soheil Sharifi[1], *Isabel Asenjo-Sanz*[1], *José A. Pomposo*[1,2,3], *Angel Alegria*[1,2]*

[1] Centro de Física de Materiales (CSIC-UPV/EHU)-Materials Physics Center (MPC), Paseo Manuel de Lardizabal 5, 20018 San Sebastián, Spain

[2]Department of Polymers and Advanced Materials: Physics, Chemistry and Technology, University of the Basque Country UPV/EHU, Paseo Manuel de Lardizabal 3, 20018 Donostia-San Sebastián, Spain

[3] IKERBASQUE-Basque Foundation for Science, Plaza de Euskadi 5, 48009 Bilbao, Spain

*E-mail: angel.alegria@ehu.eus

Table of Contents

i) Study of the enamine bond reversibility

ii) Proton NMR spectra for poly($MMA_{0.7}$-ran-$AEMA_{0.3}$)

iii) Size distribution of SCNP with different cross-link degree

iv) ATR-FTIR spectra of PMMA, AEMA, EDA and poly($MMA_{0.7}$-ran-$AEMA_{0.3}$) polymer

v) ATR-FTIR absorbance of copolymer samples with different cross-link degree

vi) Dielectric results of the crosslinked copolymers

vii) Full scale GPC chromatograms

i. Study of the enamine bond reversibility

Scheme S1 depicts the formation of two enamine bonds upon reaction of a diamine with two beta-ketoester functional groups.

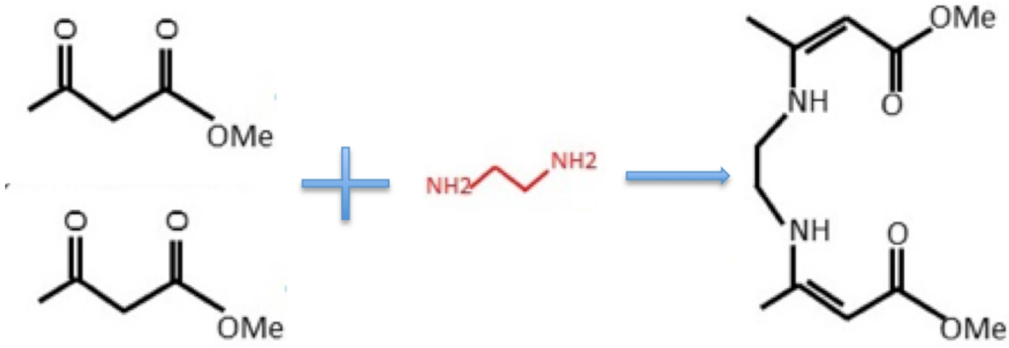

Scheme S1. Schematic view of the formation of two enamine bonds from the reaction of two units of methyl acetoacetate and diethyl amine.

The reversibility of enamine bond was previously investigated by some of us [1] as well as the synthesis and characterization of SCNPs based on intra-chain formation of enamine bonds, including extensive ^{1}H NMR and GPC characterization. In Scheme S2 we summarize the experiments that we have carried out to illustrate the reversible, dynamic nature of the enamine bonds, including treatment with a monoamine and with phosphoric acid, whereas Figures S1-S4 show the corresponding GPC results.

Moreover, to demonstrate the utility of the reversible cross-linking we have extended the treatment with phosphoric acid to the INTRA and INTER films in THF and observed that they dissolved completely. Experiments are currently under way to investigate the recyclability possibilities of these materials, that will be reported in due time.

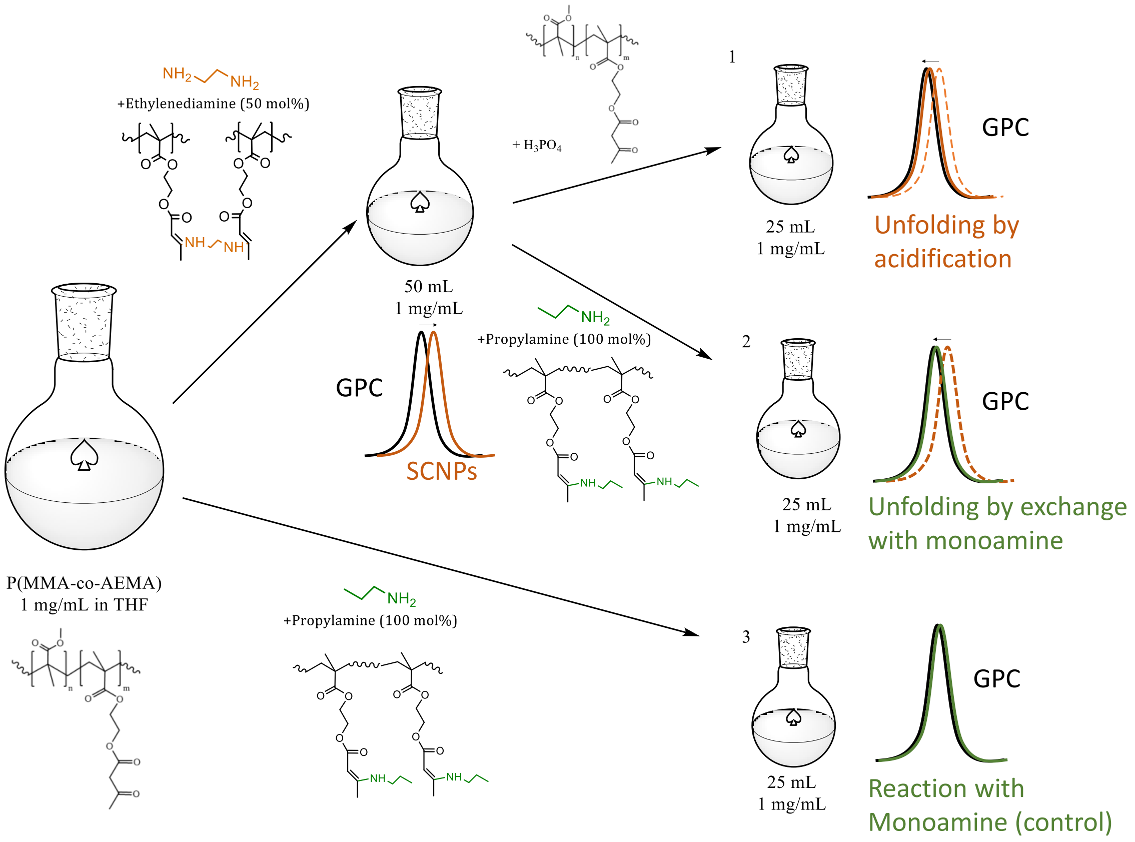

Scheme S2. Experiments carried out to illustrate the reversible, dynamic nature of the enamine bond. Addition of ethylenediamine to poly($MMA_{0.7}$-ran-$AEMA_{0.3}$) polymer at high dilution gives to the synthesis of SCNPs via enamine bond formation as revealed by GPC (see Fig. S1). Upon treatment of the SCNPs with phosphoric acid (route 1) unfolding takes place, as determined by GPC (Fig. S2). Unfolding can be also induced by exchange with a monoamine (route 2) as revealed by GPC (Figs. S3 and S4). In fact, the GPC trace upon exchange with a monoamine is identical to that recorded for the raw copolymer after reaction with the monoamine as a control experiment (route 3).

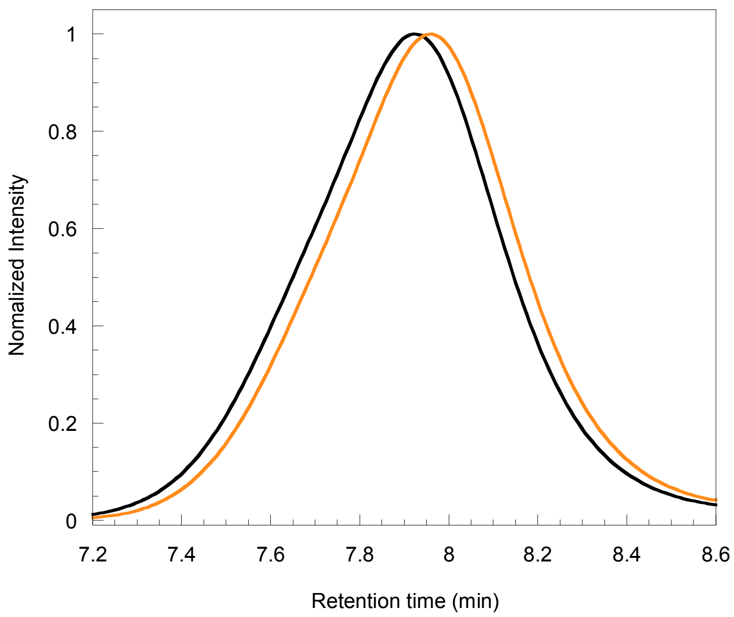

Figure S1. GPC traces of the neat poly($MMA_{0.7}$-ran-$AEMA_{0.3}$) polymer (black trace) and the SCNPs synthesized at high dilution via intra-chain enamine bond formation (orange trace).

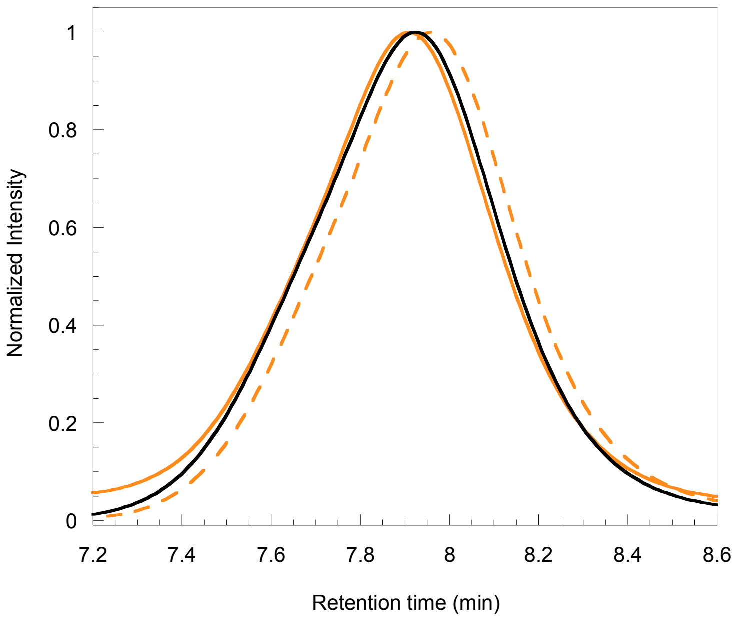

Figure S2. GPC traces of the neat poly($MMA_{0.7}$-ran-$AEMA_{0.3}$) polymer (black trace), the SCNPs synthesized at high dilution via intra-chain enamine bond formation (dashed orange trace) and the SCNPs unfolded by addition of phosphoric acid (continuous orange trace) according to route 1 in Scheme 2.

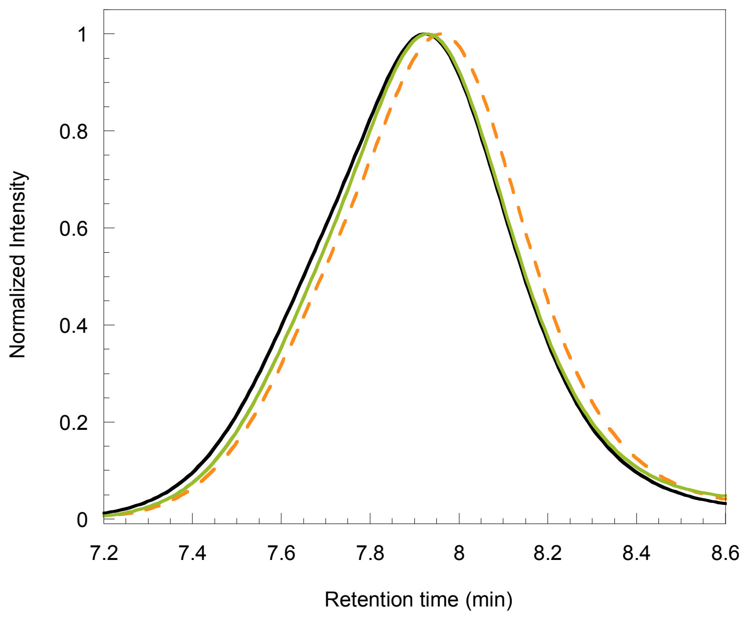

Figure S3. GPC traces of the neat poly($MMA_{0.7}$-ran-$AEMA_{0.3}$) polymer (black trace), the SCNPs synthesized at high dilution via intra-chain enamine bond formation (dashed orange trace) and the SCNPs unfolded by addition of monoamine (continuous green trace) according to route 2 in Scheme 2.

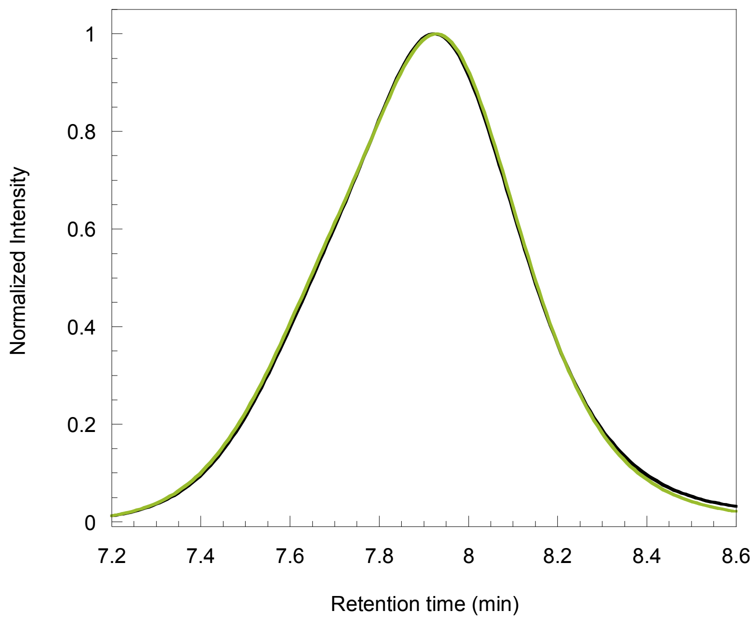

Figure S4. GPC traces of the neat poly($MMA_{0.7}$-ran-$AEMA_{0.3}$) polymer (black trace) and the copolymer functionalized with monoamine (green trace) as a control reaction according to route 3 in Scheme 2.

ii. Proton NMR spectra for poly(MMA0.7-ran-AEMA0.3)

A Bruker spectrometer was used to record ^{1}H nuclear magnetic resonance (NMR) spectra at 300MHz. The ^{1}H-NMR spectrum of the prepared copolymer solution (10mg, 0.7mL of $CDCl_3$) shown in Figure S5 allowed evaluating the chemical composition of the copolymer. The molar percentage of AEMA repeating unit in the copolymer (30±1%) was found from the analysis of the areas of peaks at 4.2 ppm and 3.6 ppm in Figure S5 using equation S1.

$$(AEMA)\% = \frac{I_a/2}{I_a/2 + \frac{(I_{b,c} - I_a)}{3}} \times 100 \qquad \text{(eq. S1)}$$

Where I_a is the area under the characteristic signal of an ethyl group of the AEMA unit and I_b is corresponding to the superposition of contributions from a methyl group of the MMA unit and a methylen group of the AEMA unit (see inset in Fig S5).

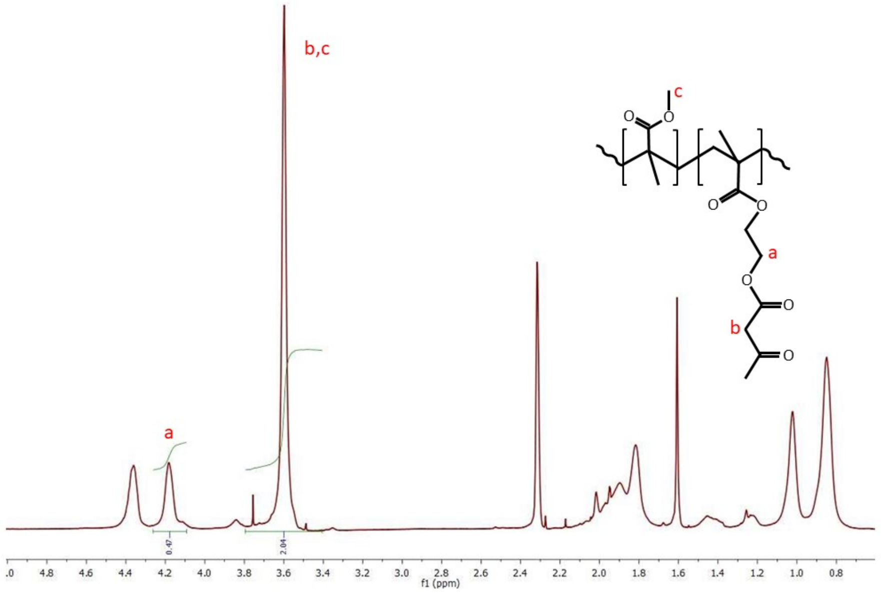

Figure S5. ^{1}H-NMR spectrum of poly($MMA_{0.7}$-ran-$AEMA_{0.3}$) in deuterated chloroform ($CDCl_3$).

iii. Size distribution of SCNP with different cross-link degree

The size distributions of single collapsed chains in dilute THF solutions (10%) were determined at room temperature, by a Malvern Zeta-sizer apparatus with scattering angle 173° and wavelength 633 nm, so that the Q-value was 0.00208 $Å^{-1}$. The light scattering curves show a peak at around 4 nm and an average size reduction by increasing the cross-linking degree, Fig. S6. This evidences that intramolecular collapse induced by cross-linking has happened after the reactions.

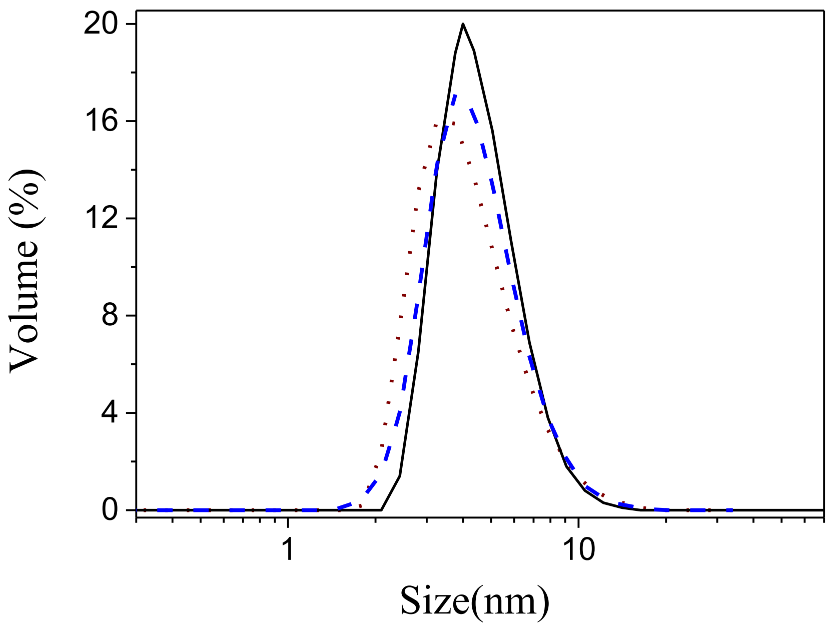

Figure S6. Size distribution from dynamic light scattering experiments of NEAT (Solid line), INTRA9 (Dashed line) and (c) INTRA50 (Dotted line).

iv. ATR-FTIR spectra of PMMA, AEMA, EDA and poly(MMA0.7-ran-AEMA0.3) copolymer

FTIR spectroscopy has been used to monitor the presence of the new enamine chemical bonds after crosslinking reaction. Experiments were done using a Jasco 3600 FT-IR Spectrometer (4 cm^{-1} resolution) in combination with an attenuated total reflection (ATR) device Golden Gate™ Single Reflection Diamond, by accumulating 50 consecutive scans. In this way ATR-FTIR spectra of pure PMMA, AEMA, EDA and the copolymer were obtained, Fig. S7. The samples have a series of characteristic IR bands, that the most intense bands are the $-CH_3$ and $-CH_2-$ stretching (~3100–2900 cm^{-1}), the carbonyl -C=O stretching (~1670–1770 cm^{-1}) and the C-O-C symmetric stretching (~1060-1200 cm^{-1}).

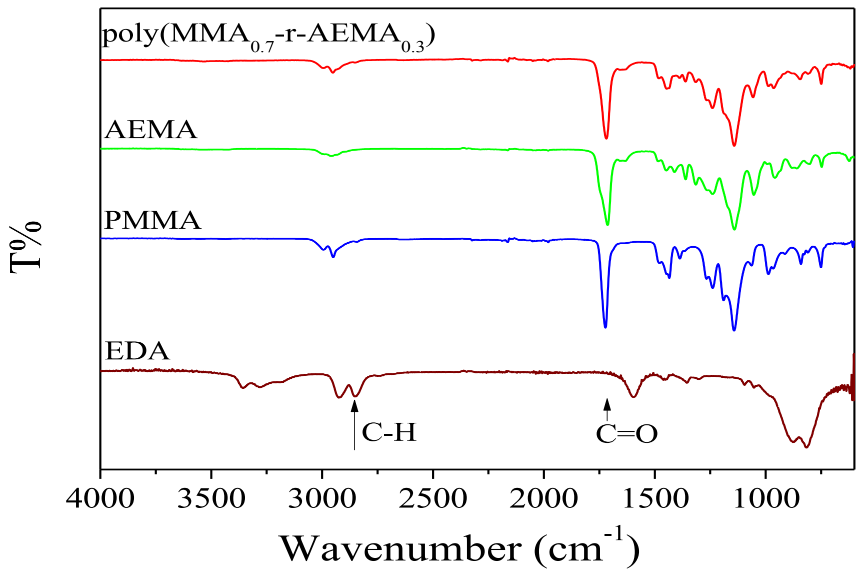

Figure S7. ATR-FTIR spectra of the PMMA, AEMA, EDA and poly($MMA_{0.7}$-ran-$AEMA_{0.3}$) copolymer

v. ATR-FTIR absorbance of copolymer samples with different cross-link degree

ATR-FTIR spectra of the copolymers in the range around 1600 cm^{-1} for different degree of cross-linking are presented in the figure S8. In the cross-linked samples, two new bands at 1656 and 1605 cm^{-1} appeared, coming from stretching vibrations of enamine bonds [1], with increasing absorption with the crosslinking density.

A quantitative evaluation was made by integration of the area under the enamine vibrational absorption band (1605–1656 cm^{-1}), Fig. S8. The baseline was considered as a straight line between 1560 and 1680 cm^{-1} and the area under the peaks calculated by numerical integration. The maximum cross-linking degree (ϕ_{max}) was calculated from number of corss-linker compare to the AEMA number. A linear relation between area under of peaks as function of maximum cross-linking degree can be observed at low value of ϕ_{max}, Fig.S8 (inserted figure) and the react cross-linking degree (ϕ) can be found from extrapolated of linear regime.

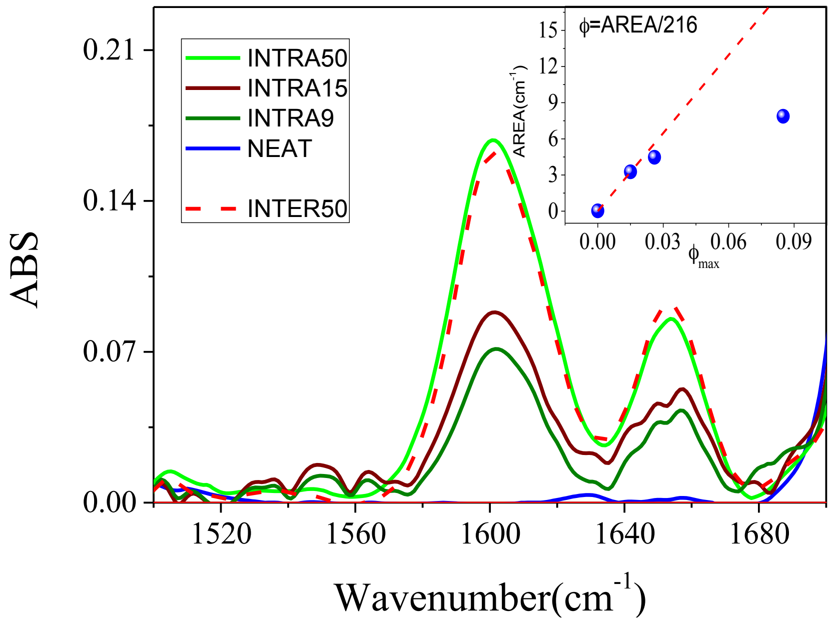

Figure S8. ATR-FTIR absorbance of poly($MMA_{0.7}$-ran-$AEMA_{0.3}$) with different cross-link degree. The insert figure is the area under the peaks calculated between 1560 and 1680 cm^{-1}. Pure poly($MMA_{0.7}$-ran-$AEMA_{0.3}$), NEAT, (Blue line), cross-linked copolymers with EDA/AEMA ratio percent from x=9 to 50, solid lines: INTRAx and dashed line: INTER50.

vi. Dielectric results on the crosslinked copolymers

Representative dielectric loss spectra of intra-chain cross-linking samples with different EDA/AEMA molar ratios obtained isothermally, are shown in Fig. S9. Similar results on inter-chain cross-linking are presented in Fig. S10.

Figure S11 shows the dielectric β- and γ-relaxation peak relaxation times determined for all the materials investigated. The Arrhenius behavior of the secondary relaxations was described with the Eyring equation (Eq. 1 in the main text). The results of this analysis indicated that the ΔS value of the β-process is reduced from 58.72 J/mol/K to 31.58 J/mol/K whereas the change in ΔH with increasing of cross-liking density is relatively small, from 83.2 kJ/mol to 71.7 kJ/mol. The ΔS reduction is indicative of a reduction of cooperativity degree.

The effect of cross-linking on the whole dielectric strength of αβ-process (Δε) as function of temperature, as obtained by the analysis of the merging of the α- and β-relaxations (main text), is presented in Figure S12. An evident increase of Δε with cross-linking density is found, whereas there is not significant effect of temperature beyond the typical uncertainties involved in the Δε determination (~ 5%).

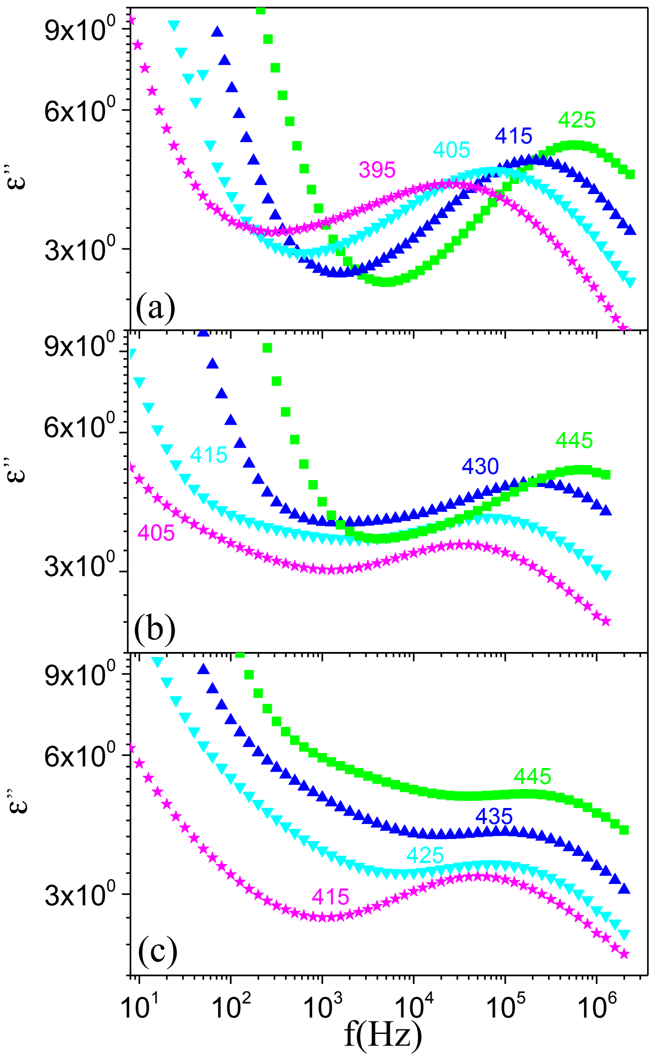

Figure S9. Dielectric permittivity losses (ε″) as function of frequency for the intra-chain cross-linking at different temperatures and different EDA/AEMA molar ratios 9%(a), 15%(b), 50%(c).

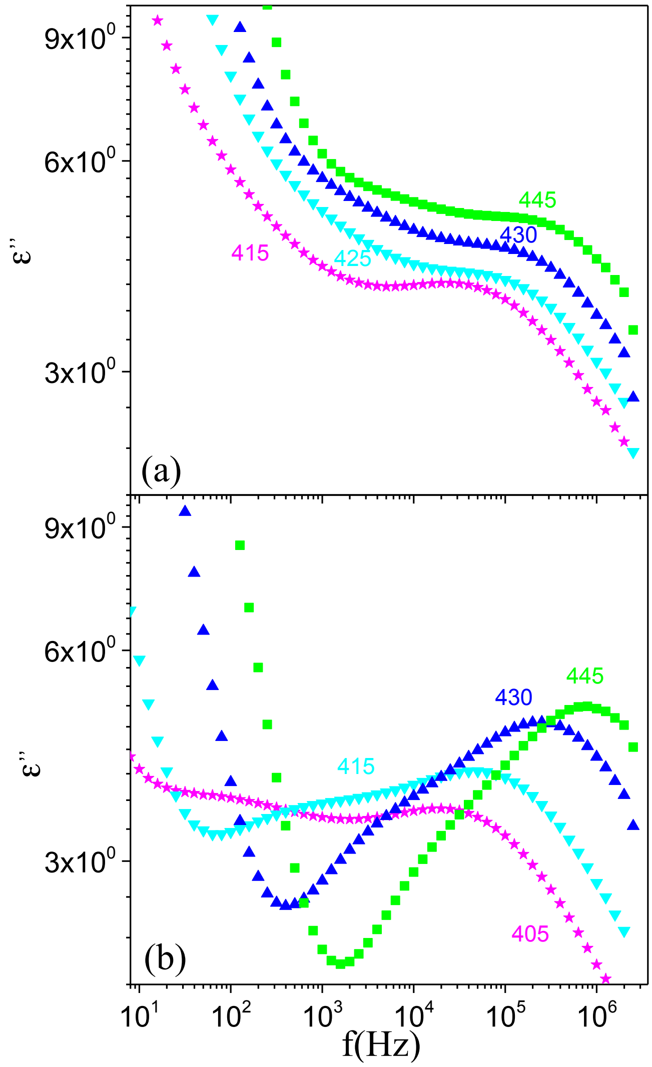

Figure S10. The dielectric permittivity losses (ε″) as function of frequency for the inter-chain cross-linking at different temperatures and different EDA/AEMA molar ratios 50%(a), 15%(b).

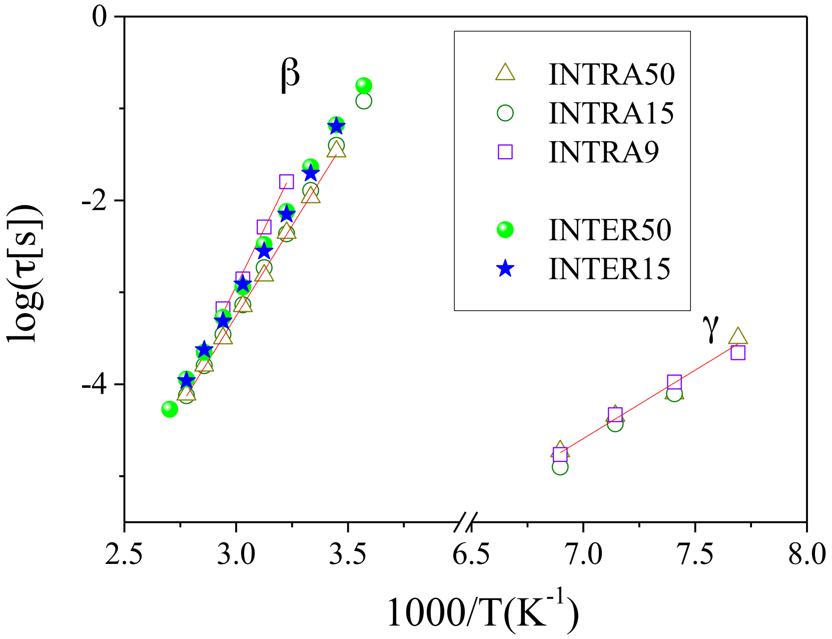

Figure S11. Temperature dependences of β- and γ-relaxation times for different cross-linking density. INTRA9 (open square symbols), INTRA15 (open circle symbols), INTRA50 (open up-triangle symbols), INTER15 (filled star symbols) and INTER50 (filled circle symbols)

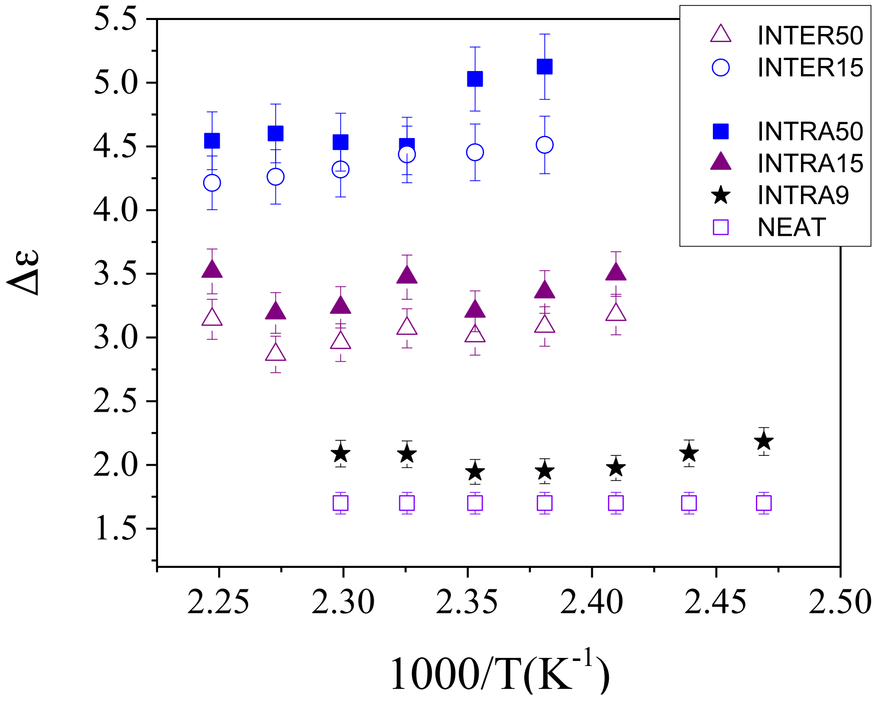

Figure S12. Temperature dependence of Δε for NEAT (open square symbols), INTRA9 (close down-triangle symbols), INTRA15 (close circle symbols points), INTRA50 (close up-triangle symbols), INTER15 (open circle symbols) and INTER50 (open up-triangle symbols). The error bars represent the typical uncertainties involved in the Δε determination (~ 5%).

vii. Full scale GPC chromatograms

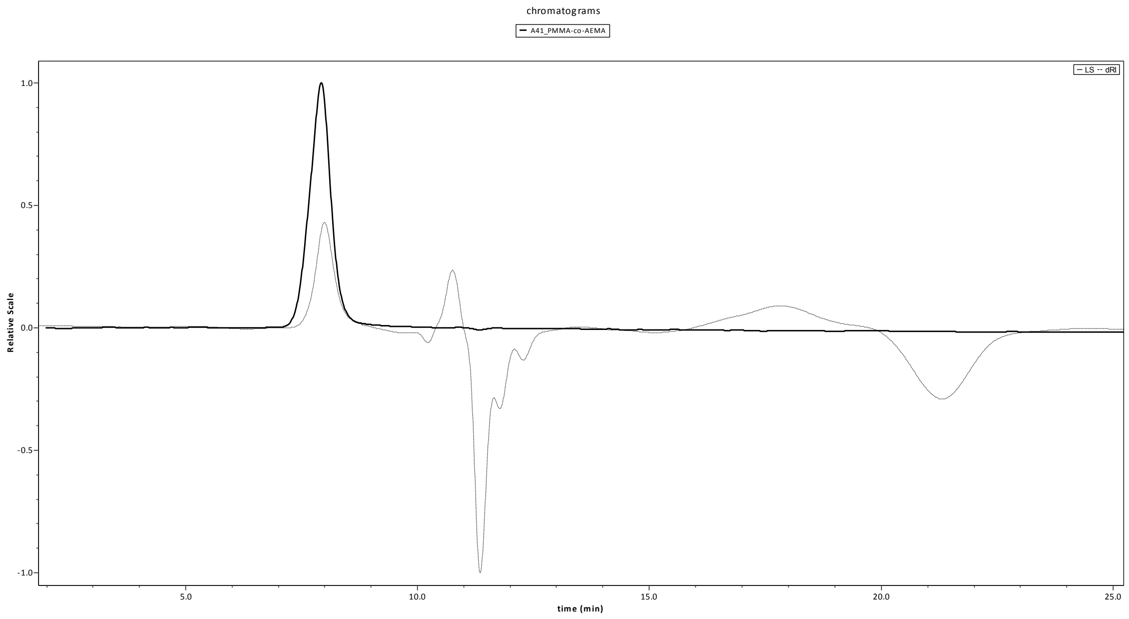

Figure S13. GPC traces (MALS and dRI detectors) of neat P(MMA-coAEMA) copolymer.

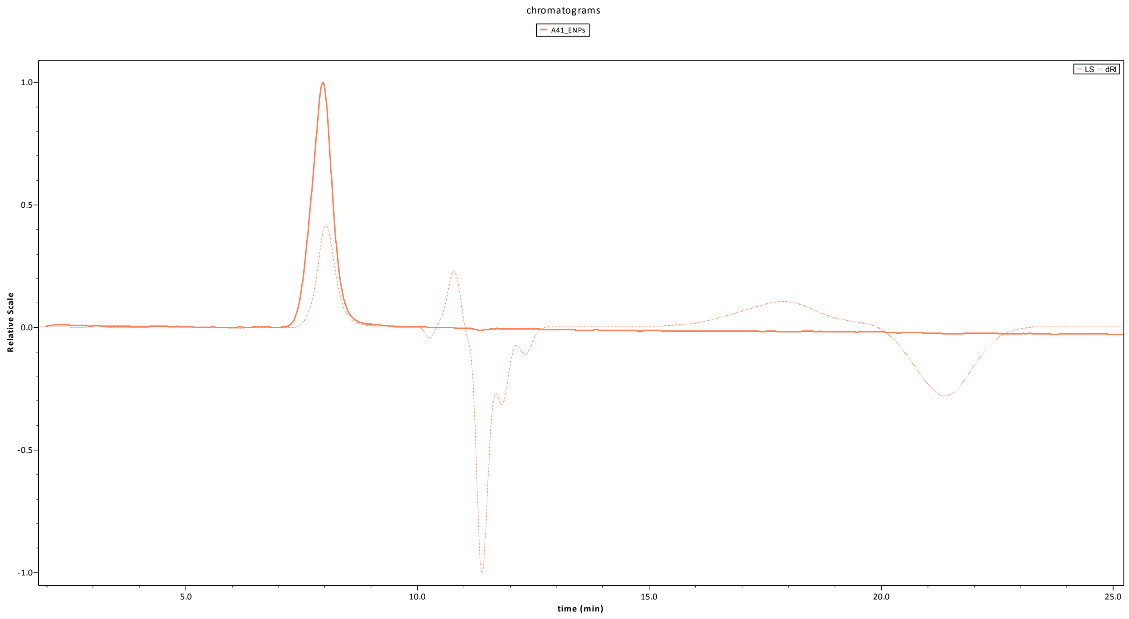

Figure S14. GPC traces (MALS and dRI detectors) of the SCNPs synthesized at high dilution via intra-chain enamine bond formation.

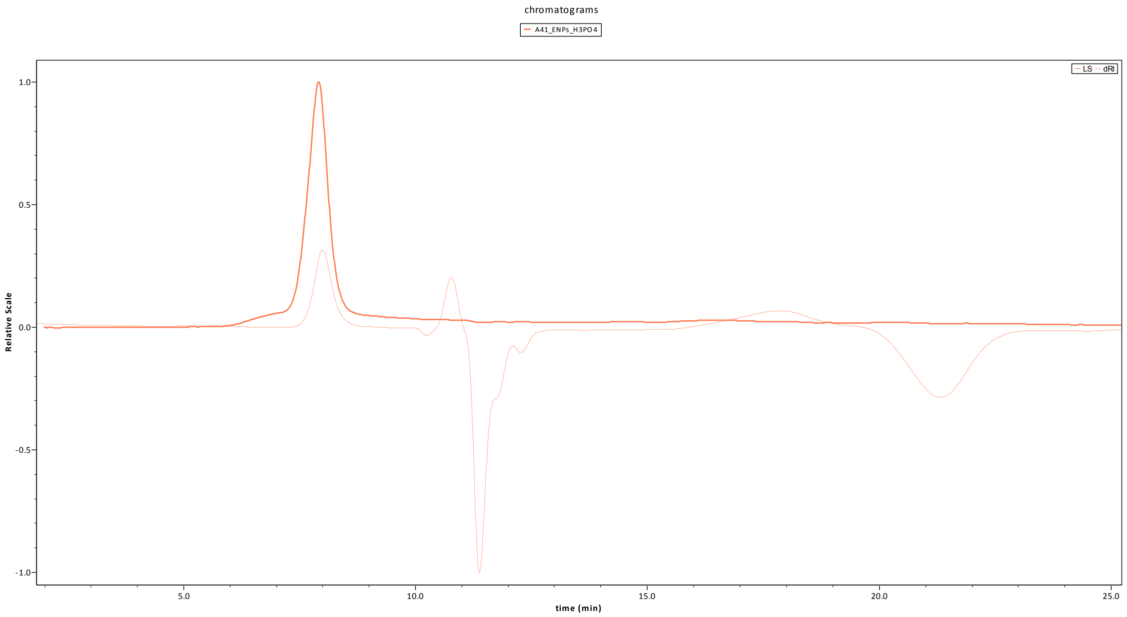

Figure S15. GPC traces (MALS and dRI detectors) of the SCNPs unfolded by addition of phosphoric acid.

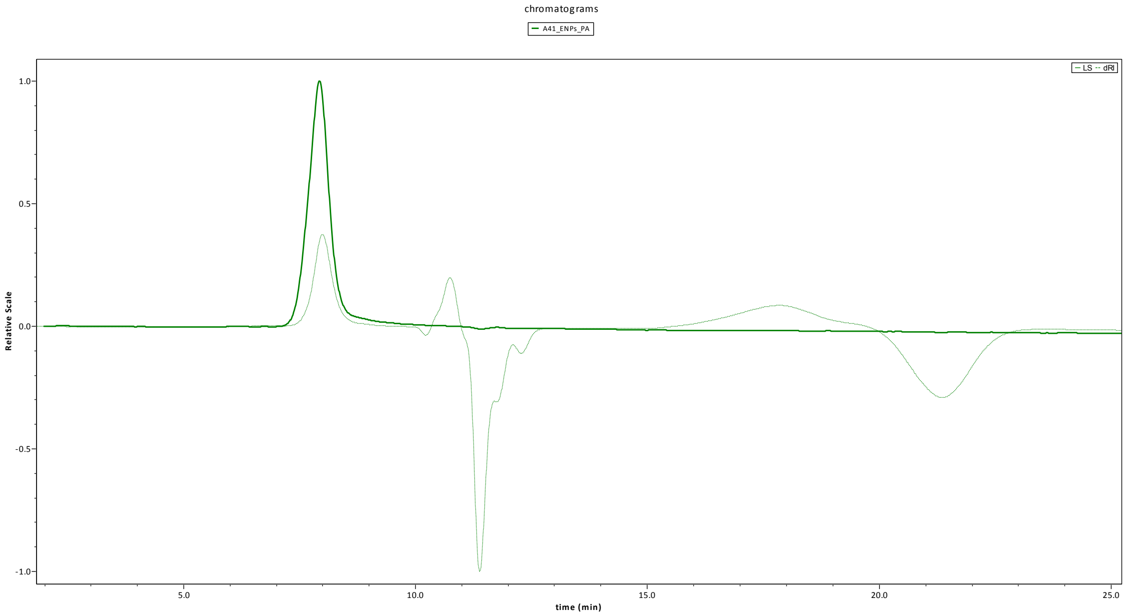

Figure S16. GPC traces (MALS and dRI detectors) of the SCNPs unfolded by addition of monoamine.

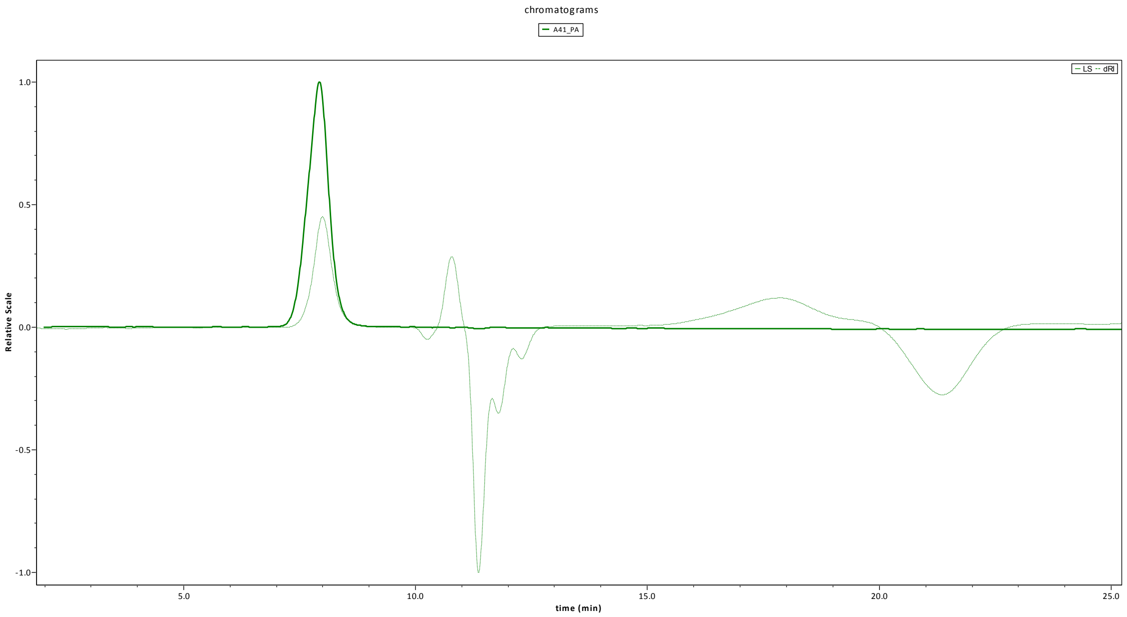

Figure S17. GPC traces (MALS and dRI detectors) of the copolymer functionalized with monoamine (green trace) as a control reaction**.**

References

[1] A. Sanchez-Sanchez, D. A. Fulton, J. A. Pomposo, pH-Responsive Single-Chain Polymer Nanoparticles Utilising Dynamic Covalent Enamine Bonds, Chem. Commun. 2014, 50, 1871-1874.
